# Fecal Detection of Calprotectin Subunits Links Inflammatory Bowel Disease Activity With Chronicity of Intestinal Inflammation

**DOI:** 10.1053/j.gastro.2025.08.040

**Published:** 2025-11-26

**Authors:** Almina Jukic, Richard Hilbe, Luis Zundel, Peter Willeit, Klaus Faserl, Christina Plattner, Andreas Zollner, Moritz Meyer, Kerstin Siegmund, Victoria Klepsch, Valentin Marteau, Arnau Vich Vila, Julian Schwärzler, Kathrin Vouk, Anna Kozsar, Dietmar Rieder, Amos Weichberger, Bettina Sarg, Felix Grabherr, Lisa Mayr, Patrizia Moser, Niloofar Nemati, Sabine Scholl-Bürgi, Daniela Karall, Georg F. Vogel, Lina Welz, Denise Aldrian, Robert Koch, Alexandra Pfister, Qitao Ran, Arthur Kaser, Richard S. Blumberg, Ivan Tancevski, Felix Sommer, Petra Bacher, Stefan Schreiber, Philip Rosenstiel, Konrad Aden, Gottfried Baier, Latifa Bakiri, Thomas Müller, Günter Weiss, TRR241 IBDome Consortium, Rinse K. Weersma, Zlatko Trajanoski, Erwin F. Wagner, Herbert Tilg, Timon E. Adolph

**Affiliations:** 1 Department of Internal Medicine I, Gastroenterology, Hepatology, Endocrinology & Metabolism, Medical University of Innsbruck, Innsbruck, Austria;; 2 Department of Internal Medicine II, Infectious Diseases, Immunology, Rheumatology, Pneumology, Medical University of Innsbruck, Innsbruck, Austria;; 3 Institute of Clinical Epidemiology, Public Health, Health Economics, Medical Statistics and Informatics, Medical University of Innsbruck, Innsbruck, Austria;; 4 Department of Public Health and Primary Care, University of Cambridge, Cambridge, United Kingdom;; 5 Biocenter, Protein Core Facility, Institute of Medical Biochemistry, Medical University of Innsbruck, Innsbruck, Austria;; 6 Biocenter, Institute of Bioinformatics, Medical University of Innsbruck, Innsbruck, Austria;; 7 Institute of Cell Genetics, Medical University of Innsbruck, Innsbruck, Austria;; 8 Department of Gastroenterology and Hepatology, University of Groningen and University Medical Center of Groningen, Groningen, the Netherlands;; 9 Biocenter, Bioinformatics Core Facility, Medical University of Innsbruck, Innsbruck, Austria;; 10 Institute of Clinical Molecular Biology, Christian Albrecht University Kiel and Schleswig-Holstein University Hospital, Kiel, Germany;; 11 Institute of Immunology, Christian Albrecht University Kiel and Schleswig-Holstein University Hospital, Kiel, Germany;; 12 INNPATH, Innsbruck Medical University Hospital, Innsbruck, Austria;; 13 Department of Pediatrics I, Medical University of Innsbruck, Innsbruck, Austria;; 14 Institute of Cell Biology, Medical University of Innsbruck, Innsbruck, Austria;; 15 Department of Cell Systems and Anatomy, University of Texas Health San Antonio, San Antonio, Texas;; 16 Cambridge Institute of Therapeutic Immunology and Infectious Disease, Department of Medicine, University of Cambridge, Cambridge, United Kingdom;; 17 Gastroenterology Division, Department of Medicine, Brigham and Women’s Hospital, Harvard Medical School, Boston, Massachusetts;; 18 Department of Laboratory Medicine, Medical University of Vienna, Vienna, Austria;; 19 Department of Dermatology, Medical University of Vienna, Vienna, Austria

**Keywords:** Calprotectin, S100A8, S100A9, Inflammatory Bowel Diseases, Crohn’s Disease, Ulcerative Colitis, Intestinal Inflammation

## Abstract

**BACKGROUND & AIMS:**

Quantification of the human S100A8/S100A9 tetrameric protein complex in stool, referred to as fecal calprotectin, is an extensively validated biomarker supporting the diagnosis and management of gastrointestinal diseases. Here, we studied the quaternary protein structures (termed configuration) of S100A8 and S100A9 and their biological function in inflammatory bowel diseases (IBD).

**METHODS:**

We dissected fecal S100A8 and S100A9 configurations in patients with IBD by size-exclusion chromatography coupled with tandem mass spectrometry and systematically defined human S100A8 and S100A9 homodimer functions compared with the calprotectin heterotetramer (CP) in the intestine of mice and in human epithelium and T cells. Moreover, we report a protein interaction network of fecal S100A8 and S100A9 in IBD.

**RESULTS:**

Stool from patients with active IBD contained abundant S100A8 and S100A9 dimers besides CP. Fecal S100A9 detection associated with clinical and endoscopic disease activity in IBD patients with low CP concentration. Oral exposure to human recombinant S100A8 and S100A9 homodimers, but not to CP, worsened intestinal inflammation in toxic and genetic mouse models. Functional profiling revealed that human S100A8 and S100A9 homodimers enhanced activation of cluster of differentiation 4^+^ and 8^+^ T cells, which promoted experimental colitis. In turn, genetic inactivation of *S100a9* protected against experimental enteritis and colitis, and pharmacologic inhibition of S100A9 ameliorated chronic colitis.

**CONCLUSIONS:**

Collectively, this study links the detection of fecal S100A9 dimers with clinical and endoscopic disease activity in IBD and identifies inflammatory actions of S100A8 and S100A9 homodimers in the intestine. Our findings pave the way for novel diagnostic and therapeutic approaches in patients with inflammatory diseases of the intestine.

## Graphical Abstract

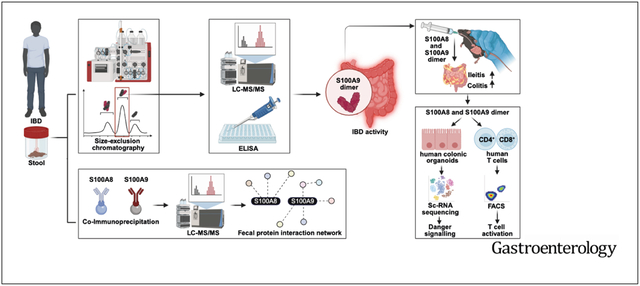

Inflammatory bowel diseases (IBD), such as Crohn’s disease (CD) and ulcerative colitis (UC), are complex immune-mediated inflammatory conditions of the intestine and extraintestinal tissues with increasing incidence and prevalence across the globe during recent decades.^1,2^ The underlying cause of chronic unresolved intestinal inflammation remains enigmatic for most patients with IBD, and host-related mediators of disease chronicity are poorly defined.^3,4^ Quantification of calprotectin (CP) in stool, determined by enzyme-linked immunosorbent assay (ELISA), is a rapid and noninvasive technique that allows the assessment of inflammatory diseases of the intestine, for example, the evaluation of suspected or established IBD.^5^ More specifically, fecal CP indicates an inflammatory condition in the intestine with high sensitivity irrespective of the underlying cause, and—in the case of IBD—mirrors endoscopic disease activity.^5^ Fecal CP concentration >150 *μ*g/g suggests active disease in patients with IBD, and normalization of fecal CP concentration (to 100–250 *μ*g/g fecal CP) is an intermediate treatment goal in IBD.^5,6^ By contrast, the biological function of CP in the human intestine is poorly understood, and experimental studies in colitis models have demonstrated conflicting biological effects.^6–9^

CP is an evolutionary conserved protein complex with ion-chelating properties. It is composed of S100A8 and S100A9 proteins that share high structural homology.^10^ In the intestine, S100A8 and S100A9 are almost exclusively expressed by myeloid cells such as neutrophils and macrophages. Upon danger signaling during infection or inflammation, S100A8 and S100A9 are passively released by dying cells^6^ or secreted by E-selectin–induced gasder-min D pores.^11^ In the extracellular space, heterotetramerization of S100A8 and S100A9 into CP is facilitated by ion availability (eg, calcium or zinc).^12^

It has been postulated that distinct quaternary protein structures (from now on referred to as *configurations*) of S100A8 and S100A9 exert divergent biological actions. For example, human S100A8 and S100A9 homodimers, but not the S100A8/S100A9 heterotetramer (CP), induced tumor necrosis factor-*α* production in monocytes.^13^ In mice, *S100a9* gene inactivation decreased experimental autoreactive cluster of differentiation (CD) 8^+^ T cells^14^ and modulated psoriasis-like inflammation,^15,16^ supporting the inflammatory nature of S100A9-containing complexes in experimental models beyond the intestine.

Here, we study the protein configurations of S100A8 and S100A9 in human stool from patients with IBD, systematically define their biological actions on human intestinal epithelium and T cells, and delineate inflammatory mechanisms in the gut of mice. We report abundant S100A9 dimers in stool from patients with active IBD, which associates with clinical and endoscopic disease activity, and that homodimers act in an inflammatory manner in the intestine by inducing cytokine responses from human colonic epithelium and by enhancing human CD4^+^ and CD8^+^ T-cell activation.

## Materials and Methods

### Human Studies

The IBDome cohort comprises patients from a multicenter phenotyping approach of IBD patients from Germany, with patient characteristics summarized in Supplementary Table 1. The phenotypic core and the related transcriptional landscape, as assessed by bulk RNA sequencing in patients with IBD and matched controls, is described as a part of an independent manuscript.^17^ Protein studies on fecal CP were performed in IBD patients at the Gastroenterology Outpatient Clinic of the Department of Internal Medicine, Medical University of Innsbruck, who were diagnosed by clinical, endoscopic, and histopathologic means with a disease duration >3 months and who provided informed consent to analyze clinical and biochemical parameters. Age-matched healthy volunteers (n = 34), with no history or biochemical evidence of gastrointestinal disease, served as controls. Investigations were performed in accordance with the Medical University of Innsbruck Ethics Committee (AN4994).

Stool samples from 316 patients from the 1000IBD cohort in Groningen, Netherlands,^18^ as well as 84 fecal aspirates from an independent IBD cohort in Kiel, Germany (AZD489/14) were analyzed, with patient characteristics summarized in Supplementary Tables 2–5. Clinical disease activity was determined on the basis of validated disease score indices and biochemical evidence of inflammation. CP configurations in stool were studied by size-exclusion chromatography coupled with liquid chromatography-tandem mass spectrometry (LC-MS/MS) and S100A8 and S100A9 concentration in stool was determined by ELISA as detailed in the Supplementary Materials and Methods. Moreover, we report a protein interaction network of fecal S100A8 and S100A9 in IBD after co-immunoprecipitation and LC-MS/MS.

### Mice

All mice were kept in a specific pathogen-free facility at the Medical University of Innsbruck. The following transgenic mouse strains were used on a C57BL/6J background: *S100a9*^−/−^ mice,^15^ *Gpx4*^*flox/wt*^*;Villin-Cre*^+^ (*Gpx4*^+^/^−*IEC*^),^19^ *Xbp1*^*flox/flox*^*;Villin-Cre*^+^ (*Xbp1*^−/−*IEC*^),^20^ *Il10*^−/−^ (Jackson Laboratory), and *Rag1*^−/−^ (Jackson Laboratory). *S100a9*^−/−^ mice were crossed onto *Gpx4*^+/−*IEC*^ and *Xbp1*^−/−*IEC*^ mice to obtain double-mutant mice. Genotyping was performed from genomic DNA extracted from ear biopsy specimens of the respective mouse strain. All experiments were conducted in accordance with institutional guidelines and with the approval of the relevant authorities (2021–0.209.767, 2023–0.839.372, 2024–0.019.456, and 2025–0.333.471). Experiments were performed with sex- and age-matched 7- to 9-week-old mice, unless stated otherwise. Treatment groups were assigned randomly, and treatments and dietary regimens are detailed below.

### Enteritis and Colitis Models

*S100a9*^−/−^ mice were orally exposed to 100 *μ*g of S100A8 or S100A9 or the 1:1 mix of S100A8/S100A9 dissolved in phosphate-buffered saline containing 0.1% bovine serum albumin for 4 consecutive days. A vehicle-treated mouse in the same cage served as a control.

To assess the role of human S100A8 and S100A9 in acute intestinal inflammation, we applied the following approach: First, C57BL/6J wild-type (WT) mice were treated with 2.5% dextran sodium sulfate (DSS; MP Biomedicals, 160110) to induce colitis. DSS was replaced with tap water, and mice were orally exposed to S100A8 (100 *μ*g/d), S100A9 (100 *μ*g/d), S100A8/A9 (100 *μ*g/d) or vehicle once daily for 4 consecutive days until conclusion of the experiment.

Next, DSS colitis was induced in *S100a9*^−/−^ mice using 2% DSS. To analyze the inflammatory potential of S100A8, S100A9, and S100A8/A9 during chronic colitis, *Il10*^−/−^ mice were orally gavaged with 100 *μ*g of recombinant protein daily for 7 consecutive days. Colitis severity was determined by clinical and histologic means, as previously reported.^21^

Pharmacologic inhibition of S100A9 was performed with oral gavage administration of 10 mg/kg paquinimod (MedChemExpress, HY-100442) once daily for 7 days in WT mice exposed to 2.5% DSS or for 14 days in *Il10*^−/−^ mice (in drinking water). In *Rag1*^−/−^mice, DSS colitis was induced and 100 *μ*g of human recombinant S100A8 or S100A9 was orally administered and compared with vehicle, as described above.

Moreover, the impact of human S100A8 and S100A9 on enteritis was determined: *Gpx4*^+/−*IEC*^ and *Xbp1*^−/−*IEC*^ mice (and littermate WT controls) were exposed to a polyunsaturated fatty acid-enriched Western diet for 3 months (ssniff, TD88137 plus 10% fish oil) to induce CD-like enteritis in mice,^22^ with or without exposure to 100 *μ*g of the human recombinant S100A8, S100A9, or S100A8/S100A9 (1:1 mix) for 7 consecutive days before conclusion of the experiment, whereas vehicle served as a control.

Next, *S100a9*^−/−^*/Gpx4*^+/−*IEC*^ double-mutant mice and respective controls were exposed to a polyunsaturated fatty acid-enriched Western diet for 3 months, *S100a9*^−/−^*/Xbp1*^−/−*IEC*^ double-mutant mice and respective controls were exposed to a chow diet, and enteritis was evaluated by clinical and histologic means. Molecular assays used to study enteritis and colitis phenotypes are detailed in the Supplementary Materials and Methods.

### Experimental Approach With Human Colonic Epithelium and Blood-Derived T Cells

Human differentiated colonic intestinal epithelial organoid monolayers were generated from non-IBD controls and stimulated with S100A8, S100A9, S100A8/S100A9, or vehicle, and transcriptional profiles of specialized epithelium were analyzed by single-cell RNA sequencing (10X Genomics), as detailed in the Supplementary Materials and Methods. Human T cells were purified, cultured, and stimulated from peripheral blood mononuclear cells and phenotyped by flow cytometry and antigen-reactive T-cell enrichment, as detailed in the Supplementary Materials and Methods.

### Bioinformatics and Statistical Analysis

RNA sequencing approaches and statistics of human cohorts, and specifically the IBDome cohort, were analyzed as described in the Supplementary Materials and Methods.

## Results

### Fecal Calprotectin Configurations in Inflammatory Bowel Disease

**Figure 1.**
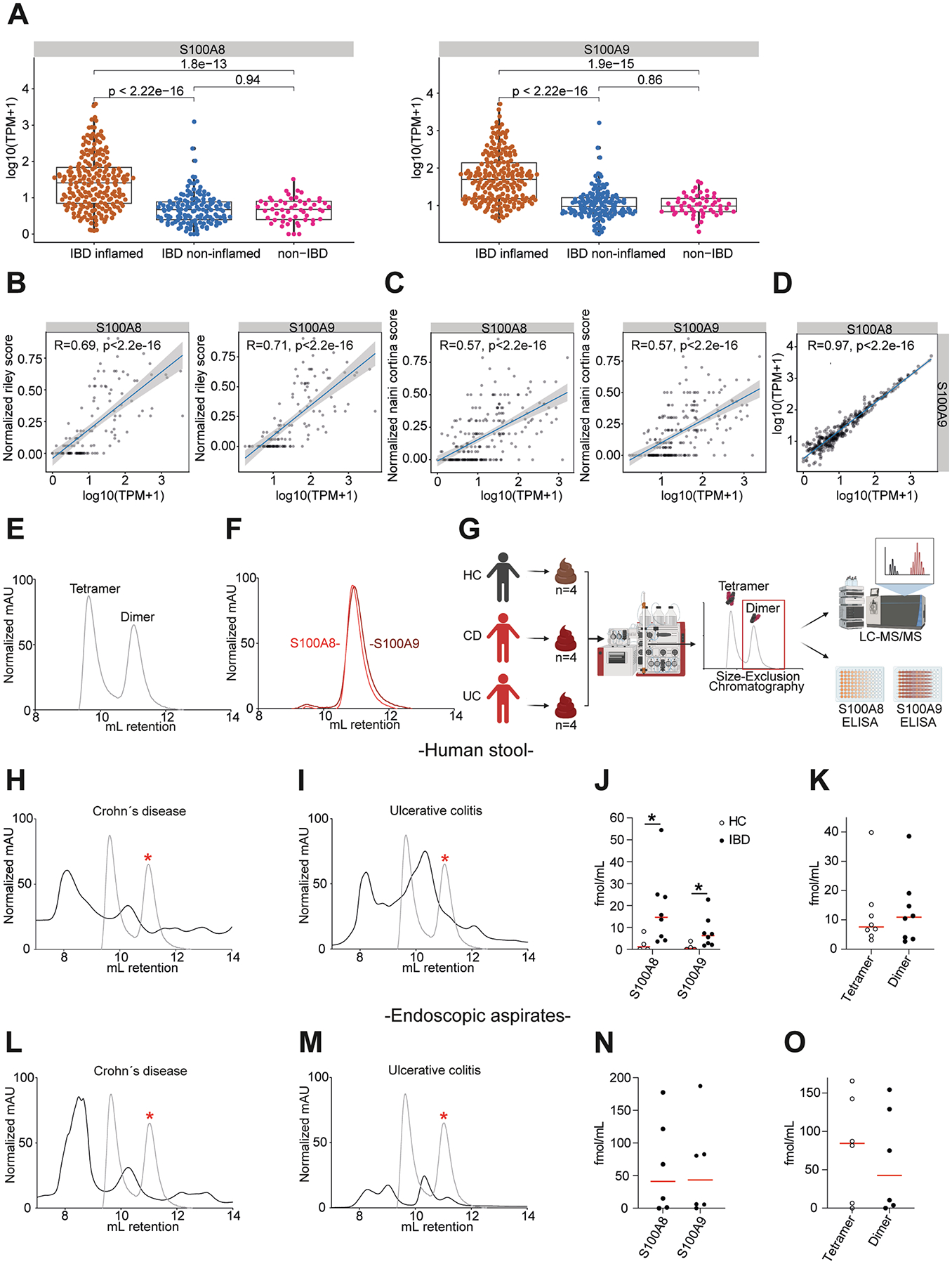
Detection of S100A8 and S100A9 dimers in stool from patients with IBD. (*A*) Box plots illustrating the mucosal expression levels of *S100A8* and *S100A9* in patients with inflamed and noninflamed IBD and non-IBD controls from the IBDome cohort, with *P* values retrieved with the Wilcoxon-Mann-Whitney test. The *boxes* indicate the 25th percentile (*bottom border*), median (*center line*), and 75th percentile (*top border*), and the *whiskers* extend to data points within 1.5* IQR (interquartile range) of the lower and upper quartiles. TPM, transcripts per million. Correlation between mucosal expression of *S100A8* and *S100A9* with histologic disease activity (*B*) in patients with UC assessed by the normalized Riley score, and (*C*) in patients with CD assessed by the normalized Naini Cortina score. (*D*) Correlation of mucosal *S100A8* and *S100A9* expression in the IBDome cohort. (*E*) Representative size-exclusion chromatography (SEC) spectra of human recombinant S100A8/S100A9 (CP), (*F*) and the overlay of human recombinant S100A8 homodimers and S100A9 homodimers. mAU, milli absorbance unit. (*G*) Schematic overview of the approach. SEC of human stool was coupled with LC-MS/MS and S100A8 and S100A9 ELISAs. The chromatography spectrum of human stool was compared with that of human recombinant protein, and fractions at a size compatible with dimers were analyzed with LC-MS/MS and S100A8- and S100A9-specific ELISA. HC, healthy control. Image was created in https://BioRender.com. (*H*) Representative SEC spectra of stool specimen dissolved in phosphate-buffered saline from patients with CD (n = 4) and (*I*) from patients with UC (n = 4). *Gray spectra* indicate chromatographic peaks of human recombinant S100A8 and S100A9 dimers indicated by a *red asterisk*. SEC fractions at this size were retrieved for detection of S100A8 and S100A9 by LC-MS/MS. (*J*) Quantification of S100A8 and S100A9 concentration in SEC fractions (of stool) compatible with homo-/heterodimers by LC-MS/MS with spike-in experiments (see Materials and Methods) (n = 4/8). (*K*) Quantification of S100A8 and S100A9 concentration in SEC fractions (of stool) by LC-MS/MS with spike-in experiments (n = 8/8). Depicted is the average of S100A8 and S100A9 concentration per patient from the SEC fraction that corresponds to the dimer fraction compared with the tetramer fraction. Representative SEC spectra of endoscopy washes from (*L*) CD patients (n = 3) and (*M*) UC patients (n = 3). (*N*) Quantification of S100A8 and S100A9 concentration in SEC fractions (of endoscopy washes) compatible with homo-/heterodimers by LC-MS/MS with spike-in experiments (n = 6/6). (*O*) Quantification of S100A8 and S100A9 concentration in SEC fractions (of endoscopy washes) by LC-MS/MS with spike-in experiments (n = 6/6). Depicted is the average of S100A8 and S100A9 concentration from the SEC fraction that corresponds to the dimer fraction, when compared with the tetramer fraction. *P < .05.

First, we assessed the transcriptional patterns of *S100A8* and *S100A9* in the human intestine by bulk RNA sequencing of tissue from the small and large intestine of 360 IBD samples (from 246 patients) compared with 54 non-IBD controls from the IBDome cohort (see Materials and Methods), with patient characteristics shown in Supplementary Table 1. *S100A8* and *S100A9* were increasingly expressed in the inflamed intestine in patients with histologic disease activity compared with noninflamed tissue from controls or IBD patients without histologic disease activity (Figure 1A and Supplementary Figure 1A). Moreover, mucosal expression of *S100A8* and *S100A9* in patients with CD and UC correlated with endoscopic (and to a lesser extent with clinical) disease activity (Supplementary Figure 1B–E) and with histologic disease activity (Figure 1B and C). Mucosal *S100A8* expression directly correlated with *S100A9* expression in patients with IBD (Figure 1D), indicating that both proteins are concomitantly expressed in the inflamed intestine, which we corroborated by immunohistochemistry (Supplementary Figure 1F).

Untargeted proteomics using liquid chromatography (LC) coupled with MS/MS (LC-MS/MS) revealed that CP is abundant in stool from patients with active UC and active CD (Supplementary Figure 1G), which enabled us to investigate protein configurations of fecal S100A8 and S100A9.

We generated human recombinant S100A8, S100A9, or the 1:1 mix (CP) (Supplementary Figure 1H), confirmed the purity (Supplementary Figure 1I–M), and then used these proteins as a reference for size-exclusion chromatography. We confirmed that the 1:1 mix of human recombinant S100A8 with S100A9 formed protein complexes at a size compatible with the calprotectin heterotetramer (CP), and to a lesser extent homo- and heterodimers, which cannot be resolved with this technique (Figure 1E). Human recombinant S100A8 spontaneously formed protein complexes at a size compatible with S100A8/S100A8 homodimers, as similarly observed for human recombinant S100A9 homodimers (Figure 1F and Supplementary Figure 1L and M).

Next, we established size-exclusion chromatography with human stool from 4 non-IBD controls and human recombinant CP as a reference (Supplementary Figure 1N) and then performed size-exclusion chromatography with stool from 8 patients with active IBD (4 active UC and 4 active CD with fecal calprotectin >500 *μ*g/g). We analyzed whether a fraction retrieved from size-exclusion chromatography at molecular sizes compatible with homo-heterodimers (as largely determined by the hydrodynamic radius) enabled detection of S100A8 or S100A9 by LC-MS/MS and by ELISA (Figure 1G).

Most notably, size-exclusion chromatography fractions from active CD and UC patients contained S100A8 and S100A9 at sizes compatible with homo-/heterodimers (Figure 1H and I), as indicated by LC-MS/MS (Figure 1J) and by ELISA (Supplementary Figure 1O). Notably, the concentration of S100A8 and S100A9 homo-/heterodimers was comparable to that of the heterotetramer (CP) in the respective size-exclusion chromatography fractions (Figure 1K). By contrast, stool from non-IBD controls did not show evidence of S100A8 and S100A9 homo-/heterodimers in the specific size-exclusion chromatography fractions (Figure 1J and Supplementary Figure 1O).

Moreover, analysis of endoscopy-retrieved fecal colonic aspirates from patients with active IBD (3 CD and 3 UC patients) validated the presence of S100A8 and S100A9 homo-/heterodimers, as indicated by LC-MS/MS and by ELISA (Figure 1L–N and Supplementary Figure 1P). The concentration of S100A8 and S100A9 homo-/heterodimers were similar to those of the heterotetramer (CP) in the respective size-exclusion chromatography fractions (Figure 1O). Collectively, these studies demonstrated that the mucosal expression of *S100A8* and *S100A9* correlated with clinical, endoscopic, and histologic disease activity and that S100A8 and S100A9 homo-/heterodimers were detectable in patients with active IBD in addition to the heterotetramer (CP).

### S100A8 and S100A9 Dimer Detection in Inflammatory Bowel Disease

**Figure 2.**
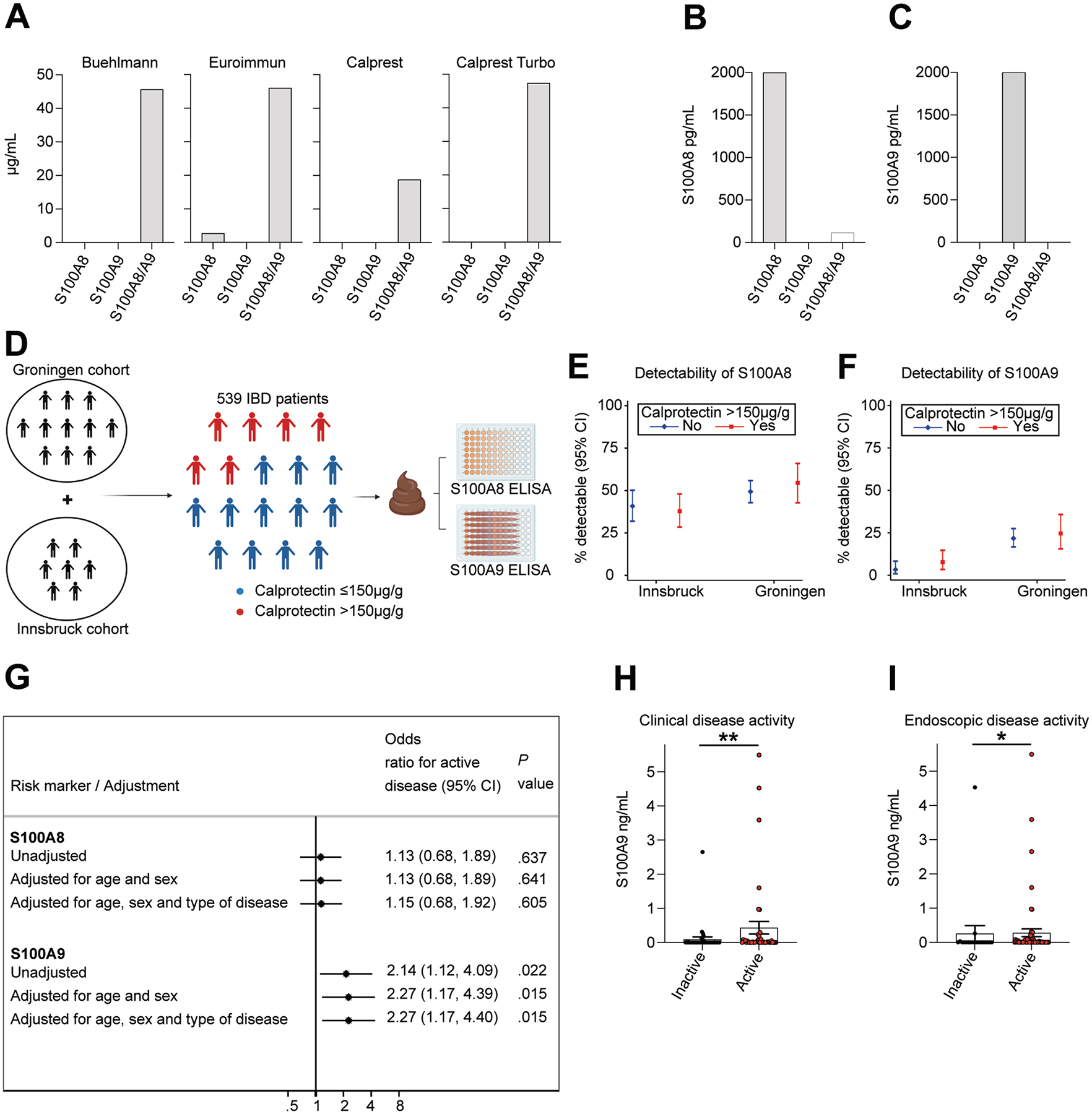
Quantification of S100A8 and S100A9 in relation to IBD activity. (*A*) Quantification of human recombinant S100A8, S100A9, and S100A8/S100A9 (CP) with indicated ELISAs used in clinical routine. Quantification of human recombinant (*B*) S100A8 and (*C*) S100A9 with commercial research ELISAs. (*D*) Schematic overview of the approach. We analyzed stool from 539 patients of 2 IBD cohorts from Innsbruck (Austria, n = 223) and Groningen (the Netherlands, n = 316). The concentration of fecal CP exceeded 150 *μ*g/g stool in 180 patients, and the remaining patients were characterized by CP levels ≤150 *μ*g/g. All stool samples were then analyzed by specific S100A8 and S100A9 ELISA. Image was created in https://BioRender.com. Percentage of IBD patients with fecal CP levels ≤150 *μ*g/g or >150 *μ*g/g from the Innsbruck and Groningen cohorts with detectable (*E*) S100A8 or (*F*) S100A9 protein in stool, as determined by ELISA (95% confidence interval [CI]). (*G*) Association of fecal detection of S100A8 or S100A9 in patients with clinical disease activity and fecal CP concentration ≤150 *μ*g/g, as determined by logistic regression modeling, with and without adjustment for age, sex, and type of disease. CI, confidence interval. Quantification of S100A9 concentration in endoscopic aspirates from the colon of IBD patients with CP concentration ≤150 *μ*g/mL in IBD patients stratified by (*H*) clinical disease activity or (*I*) endoscopic disease activity in a cohort from Kiel, Germany (n = 84) (mean ± SEM shown; Mann-Whitney *U* test). *P < .05, **P < .01.

These findings led us to assess the diagnostic utility of S100A8 and S100A9 dimer detection in IBD. Four fecal CP assays used in clinical routine neither detected human recombinant S100A8 nor S100A9 homodimers (Figure 2A), while detecting the 1:1 mix of the human recombinant S100A8/S100A9 heterotetramer (CP), indicating a diagnostic gap of current clinical assays in the detection of S100A8 and S100A9 dimers. Consequently, we used specific ELISAs (established for research use) that allowed detection of human recombinant S100A8 and S100A9 homodimers, without detecting the S100A8/S100A9 heterotetramer (CP) (Figure 2B and C), to estimate their presence and concentration in stool from patients with IBD. We analyzed the stool from 539 patients of 2 IBD cohorts from Innsbruck (Austria, n = 223) and Groningen (the Netherlands, n = 316) (Figure 2D), with patient characteristics listed in Supplementary Table 2. More specifically, 338 patients (63%) were diagnosed with CD and 201 patients (37%) with UC. CP was detectable (>16 *μ*g/g) in 413 patients (77%), and the concentration was >150 *μ*g/g stool in 180 patients (33%). Across all patients, fecal S100A8 was detectable in 248 patients (46%), with a median concentration of 5.0 ng/g, and fecal S100A9 was detectable in 83 patients (15%), with a median concentration of 5.4 ng/g, which was unrelated to fecal CP concentration (Figure 2E and F). In line, we did not note a correlation between fecal CP and S100A8 or S100A9 concentration (Supplementary Figure 2A and B). Thirty-four adults without a history of IBD and without gastrointestinal symptoms (non-IBD controls) did not exhibit detectable levels of S100A9 in stool, while 7 of 34 healthy controls exhibited minute evidence for S100A8 (Supplementary Figure 2C). These findings indicated that fecal S100A8 and S100A9 dimer detection was frequently demonstrable in patients with IBD independent from fecal CP concentration.

Fecal CP is used in clinical practice as a biomarker to help identify patients with active disease, with >150 *μ*g/g CP in stool suggesting an inflammatory condition in patients with IBD.^23^ However, symptoms compatible with active IBD are commonly reported in patients with fecal CP <150 *μ*g/g, indicating a limitation of fecal CP as a biomarker that comes along with uncertainty in clinical management.

Therefore, we next assessed the utility of S100A8 and S100A9 detection in patients with symptomatic disease (ie, clinical disease activity) and fecal CP ≤150 *μ*g/g in the cohort from Innsbruck and Groningen. Among the 356 patients with available disease activity scores and a fecal CP concentration ≤150 *μ*g/g stool, 75 (21%) had clinically active disease and 281 (79%) were in remission (Supplementary Table 2).

By logistic regression modeling (without adjustment for age, sex, type of disease), we noted that fecal detection of S100A9, but not detection of S100A8, was associated with 2.14-fold odds of having clinically active disease (95% confidence interval, 1.12–4.09; *P* = .022). The association between S100A9 and clinically active disease remained robust when adjusting for age and sex (odds ratio, 2.27; 95% confidence interval, 1.17–4.39, *P* = .015), and additionally for disease type (odds ratio, 2.27; 95% confidence interval, 1.17–4.40; *P* = .015) (Figure 2G). Neither disease location (Montreal L1-L3) nor smoking status (current smoker) associated with detection of fecal S100A8 or S100A9 dimers (Supplementary Tables 3 and 4).

We corroborated these results by comparison of endoscopy reports and clinical disease assessment with ELISA-based colonic aspirate quantification of S100A9 in 84 patients with IBD from Kiel, Germany (Supplementary Table 5). Indeed, detection of S100A9 was associated with clinical disease activity (Figure 2H) and with endoscopic disease activity (Figure 2I) in patients with low CP concentration (≤150 *μ*g/mL). Notably, detection of S100A8 in colonic aspirates was also associated with endoscopic but not clinical disease activity (Supplementary Figure 2D and E).

Collectively, these studies demonstrated that S100A8 and S100A9 were commonly detectable in the human intestine and stool of patients with IBD and that fecal S100A9 concentration was associated with clinical and endoscopic disease activity in patients with low fecal CP concentration (≤150 *μ*g/g). Larger endoscopy trials are warranted to determine the utility of S100A8 and S100A9 as biomarkers for active IBD in clinical practice.

### Human S100A8 and S100A9 Homodimers Promote Enteritis and Colitis in Mice

**Figure 3.**
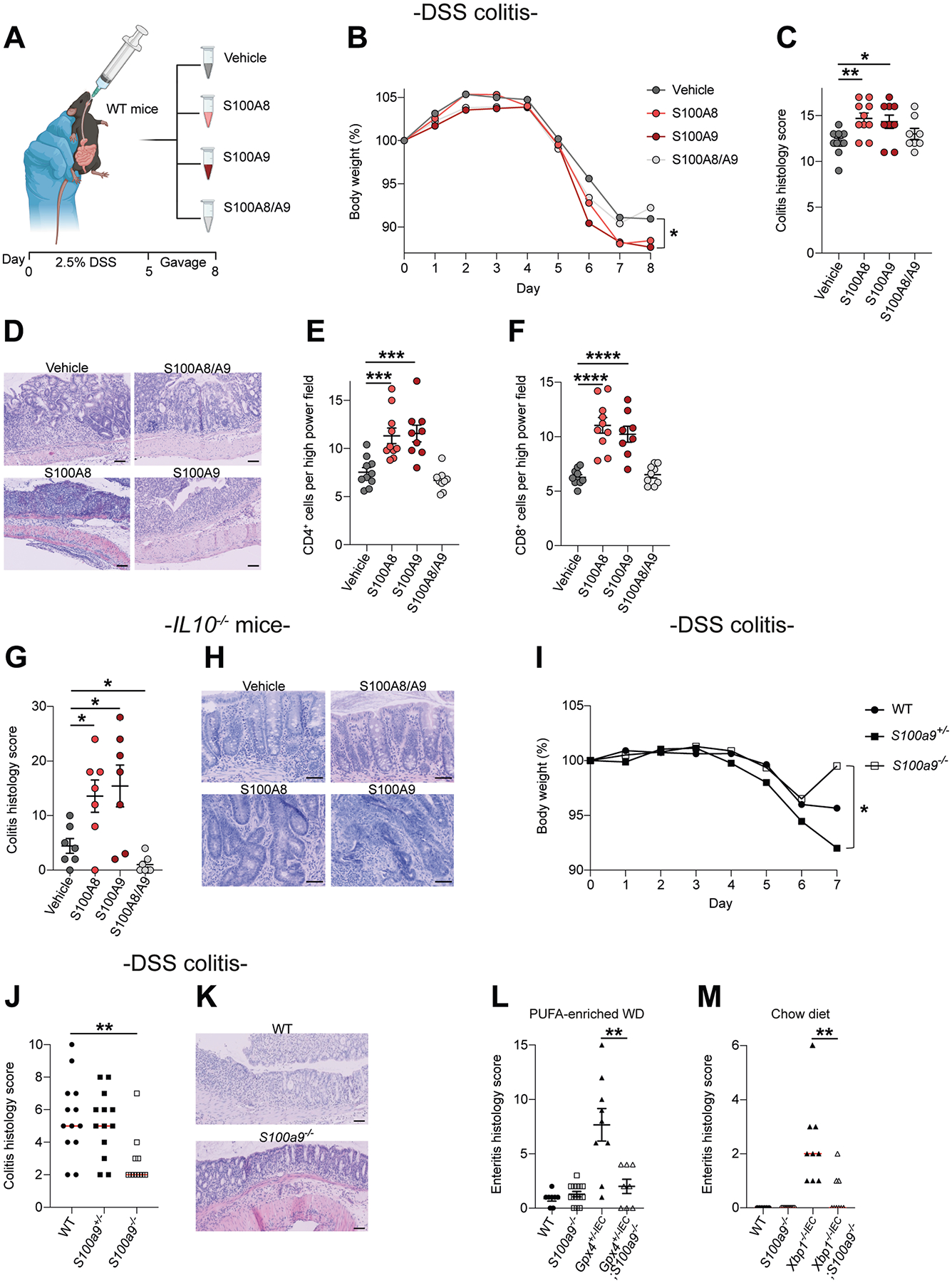
Human S100A8 and S100A9 homodimers promote enteritis and colitis in mice. (*A*) Schematic representation of approach: WT mice were exposed to 2.5% DSS for 5 days and subsequently exposed to 100 *μ*g S100A8 or S100A9, the S100A8/S100A9 1:1 mix (CP), or vehicle by gavage once daily for 4 days. Image was created in https://BioRender.com. (*B*) Body weight course of mice, with (*C*) colitis histology score at day 8 (n = 8–10; 8–9 weeks; mean ± standard error of the mean [SEM] shown; 1-way analysis of variance [ANOVA] with post hoc Bonferroni) and (*D*) representative H&E images. *Scale bars*, 100 *μ*m. Quantification of (*E*) CD4^+^ T cells and (*F*) CD8^+^ T cells in the colon of WT mice (scored in Figure 1C), as determined by immunohistochemistry (mean ± SEM shown; 1-way ANOVA with post hoc Bonferroni). (*G*) Colitis histology score and (*H*) representative H&E images of *Il10*^−/−^ mice after oral exposure to vehicle, S100A8, S100A9 or the 1:1 mix (CP) (n = 7 each; 8–9 weeks; 1-way ANOVA with post hoc Holm’s correction; mean ± SEM). *Scale bars*, 100 *μ*m. (*I*) Body weight course of mice, with (*J*) colitis histology score and (*K*) representative H&E images of WT (n = 13), *S100a9* ^+/−^ (n = 13), and *S100a9*^−/−^ (n =11) mice after 5 days of DSS (8–9 weeks; Kruskal-Wallis test with Dunn’s correction; median shown). *Scale bars*, 100 μm. (*L*) Enteritis histology score of *Gpx4* ^+/−*IEC*^ (n = 9) and *Gpx4* ^+/–*IEC*^;*S100a9*^−/−^ (n = 8) mice fed a polyunsaturated fatty acid (PUFA)-enriched Western diet (WD) for 3 months (mean ± SEM shown, 2-tailed Student *t* test). (*M*) Enteritis histology score of *Xbp1*^−/−*IEC*^ (n = 9) and *Xbp1*^−/−*IEC*^;*S100a9*^−/−^ (n = 9) mice fed a chow diet (7–8 weeks; median shown, Mann-Whitney *U* test). *P < .05, **P < .01, ***P < .001, ****P < .0001.

Because we identified luminal S100A8 and S100A9 dimers in stool and colonic aspirates from patients with IBD, we rationalized that oral gavage of human S100A8 and S100A9 dimers should enable the study of their biological effect in the mouse intestine. Indeed, oral gavage of human recombinant S100A8 and S100A9 homodimers led to distribution along the murine gastrointestinal tract and was specifically detectable in small intestinal mucosa, gut epithelium, and colonic content (Supplementary Figure 3A and B). Likewise, S100A8 and S100A9 homodimers were taken up by MODE-K intestinal model epithelium (Supplementary Figure 3C and D). We exposed WT mice to 2.5% DSS and orally gavaged S100A8 homodimers, S100A9 homodimers, the S100A8/S100A9 heterotetramer (CP), or vehicle for 4 consecutive days starting at day 5 when colitis was established (Figure 3A). S100A8 and S100A9 homodimers, but not the heterotetramer (CP), promoted DSS colitis compared with vehicle, indicated by clinical and histologic means (Figure 3B–D). S100A8 and S100A9 homodimers also promoted DSS colitis in *S100a9*^−/−^ mice (Supplementary Figure 3E and F), which lack S100A9-containing species and expressed no S100A8 in the gut (Supplementary Figure 3G and H), suggesting that the inflammatory effect of S100A8 and S100A9 homodimers could not be explained by chimeric effects of human with mouse protein in this model. Notably, S100A8 and S100A9 homodimers increased CD4^+^ and CD8^+^ T-cell infiltration in the inflamed mucosa (Figure 3E and F and Supplementary Figure 3I and J).

In line, oral gavage of S100A8 and S100A9 homodimers for 7 consecutive days, but not CP, promoted chronic colitis in *Il10*^−/−^ mice (Figure 3G and H). Moreover, S100A8 or S100A9 homodimers, but not CP, worsened experimental enteritis in *Gpx4*^+/−*IEC*^ and in *Xbp1*^−/−*IEC*^ mice (Supplementary Figure 3K and L), which was induced by a model Western diet for 3 months.^19,22^ Conversely, genetic inactivation of *S100a9* protected against experimental gut inflammation. *S100a9*^−/−^ mice were protected against DSS colitis compared with heterozygous *S100a9*^+/−^ or WT mice (Figure 3I–K). Inactivation of *S100a9* also ameliorated experimental enteritis in *Gpx4*^+/−*IEC*^ mice (induced by a model Western diet), and spontaneous enteritis in *Xbp1*^−/−*IEC*^ mice (Figure 3L and M and Supplementary Figure 3M and N). Collectively, these data demonstrated that human S100A8 and S100A9 homodimers worsened experimental enteritis and colitis in mice and, vice versa, that genetic inactivation of *S100a9* ameliorated experimental enteritis and colitis.

### Human S100A8 and S100A9 Homodimers Induce an Inflammatory Response in Intestinal Epithelium

**Figure 4.**
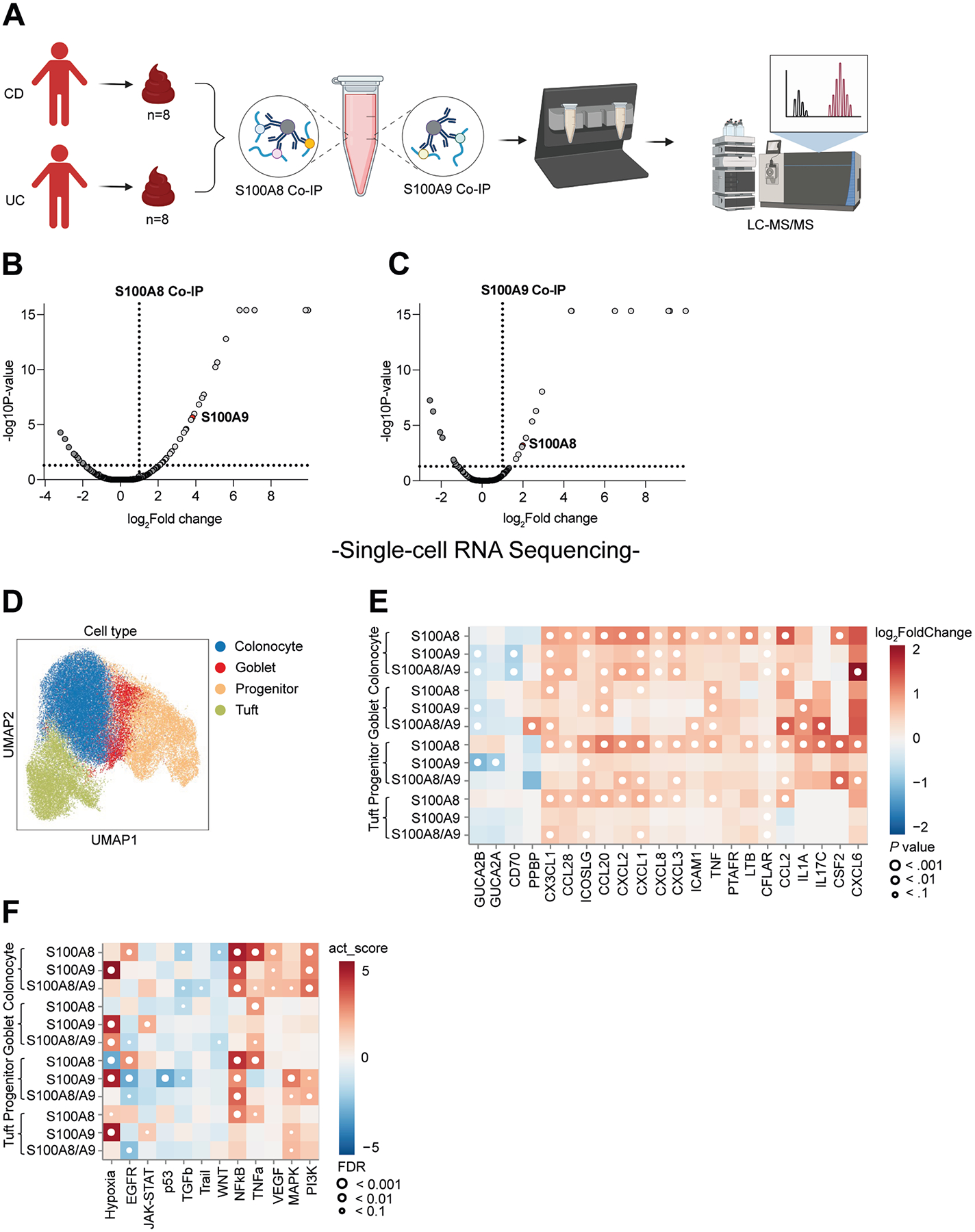
Human S100A8 and S100A9 homodimers induce epithelial cytokine responses. (*A*) Schematic representation of co-immunoprecipitation (Co-IP) experiments performed for identification of protein interaction partners of S100A8 and S100A9 in the gut lumen of IBD patients shown in *B* and *C*. Image was created in https://BioRender.com. Volcano plot depicting protein interaction partners of (*B*) S100A8 and (*C*) S100A9 in stool from patients with active IBD (CD, n = 8; UC, n = 8), as identified by LC-MS/MS analysis after Co-IP. Significant protein interactions are defined by a *P* value <.05 and a fold change of >2. (*D*) Single-cell RNA sequencing of human colonic organoids, differentiated into monolayers, with Uniform Manifold Approximation and Projection (UMAP) projection depicting epithelial cell populations. (*E*) Heat map showing differential cytokine gene expression per cell-type in differentiated organoids treated with S100A8, S100A9, or S100A8/S100A9 (1:1), relative to vehicle. Red indicates upregulation of cytokine expression, blue indicates down-regulation. (*F*) Pathway RespOnsive GENes for activity inference (PROGENy) pathway activity scores (using a multivariate linear model) per epithelial cell type in differentiated organoids exposed to S100A8, S100A9, or S100A8/S100A9 (1:1). Red indicates up-regulation of a pathway relative to vehicle. CCL, chemokine (C-C motif) ligand; CFLAR, CASP8 and FADD like apoptosis regulator; CSF2, colony-stimulating factor 2; CX3CL1, C-X3-C motif chemokine ligand 1; CXCL, chemokine (C-X-C motif) ligand; EGFR, epidermal growth factor receptor; FDR, false discovery rate; GUCA, guanylate cyclase activator; ICAM, intercellular adhesion molecule; ICOSL inducible co-stimulator ligand; IL, interleukin; LTB, lymphotoxin-beta; MAPK, mitogen-activated protein kinase; NF*κ*B, nuclear factor-*κ*B; PI3K, phosphatidylinositol-3-kinase; PPBP, pro-platelet basic protein; PTAFR, platelet-activating factor receptor; JAK/STAT, Janus kinase/signal transducer and activator of transcription; TGF, transforming growth factor; TNF, tumor necrosis factor; TRAIl, TNF-related apoptosis-inducing ligand; VEGF, vascular endothelial growth factor.

Next, we investigated inflammatory effects of human S100A8 and S100A9 homodimers. In a first step, we performed antibody-mediated immunoprecipitation of S100A8 and S100A9 dimers from stool of 16 patients with active IBD and consequently defined the fecal protein interaction network by means of untargeted LC-MS/MS (Figure 4A). S100A8 interacted with S100A9 (and vice versa) in stool from patients with active IBD (Figure 4B and C), demonstrating the validity of our approach. Several protein interaction partners of S100A8 and S100A9 in stool from active IBD patients were identified with this approach (Supplementary Tables 6 and 7), some of them suggesting actions at the intestinal epithelium (eg, interaction with galectin-4 and matrix metalloproteinase 15). Thus, we used single-cell RNA sequencing of differentiated human colonic organoids upon stimulation with S100A8 or S100A9 homodimers to determine the transcriptional response of specialized intestinal epithelium when compared with vehicle exposure (Figure 4D). Indeed, S100A8 and S100A9 homodimers broadly induced cytokine expression in differentiated colonocytes, for example, chemokine ligand 20 in goblet cells (Figure 4E). Moreover, Pathway RespOnsive GENes for activity inference (PROGENy) pathway activity analysis indicated that S100A8 homodimers induced a transcriptional tumor necrosis factor and nuclear factor-*κ*B response in colonocytes, compared with vehicle, and that S100A9 induced hypoxia signaling (Figure 4F). Collectively, these data indicated that S100A8 and S100A9 homodimers induce an inflammatory response in human intestinal epithelium.

### Human S100A8 and S100A9 Homodimers Enhance Cluster of Differentiation 4-Positive and 8-Positive T-Cell Activation

**Figure 5.**
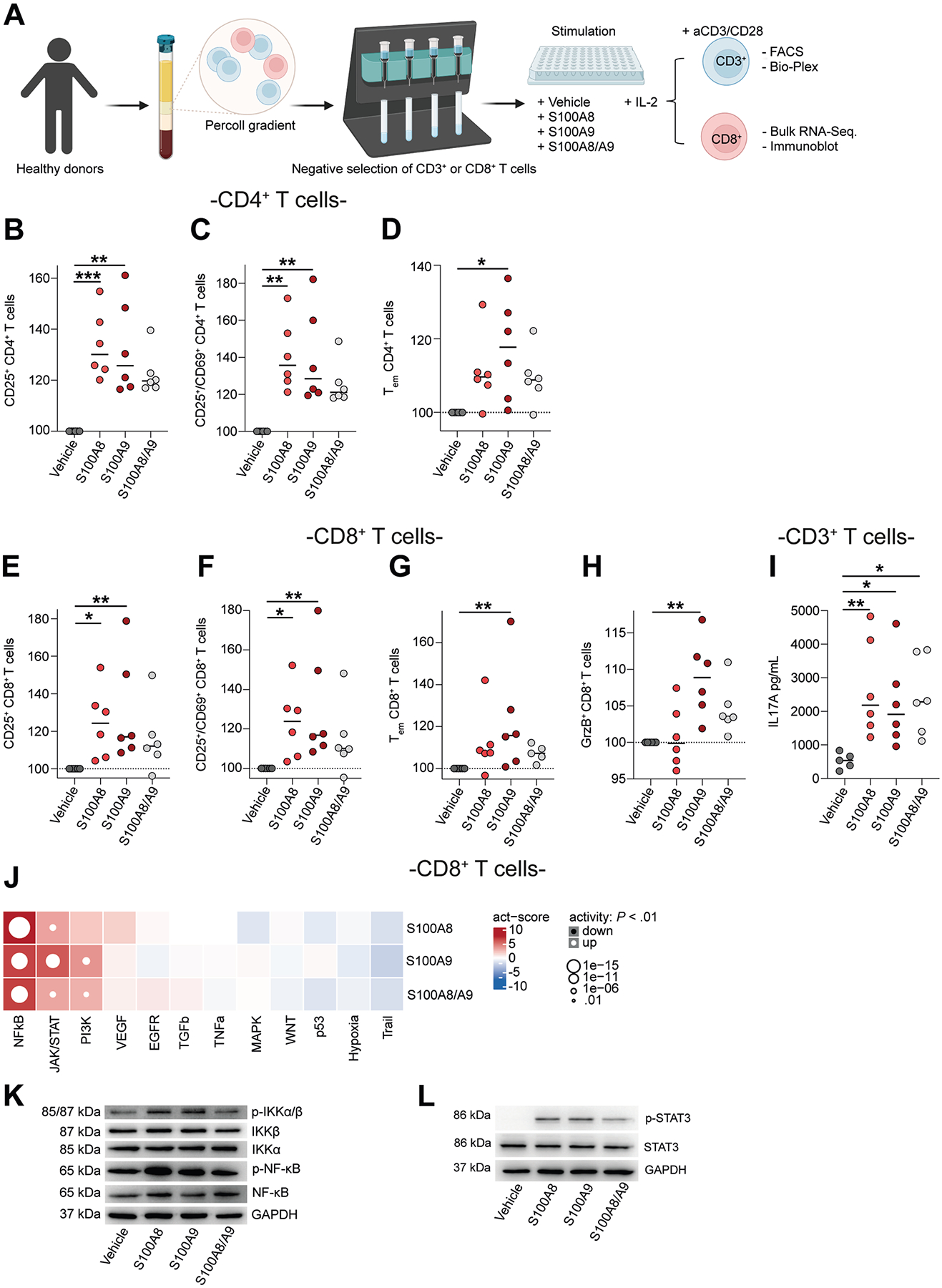
Human S100A8 and S100A9 homodimers enhance T-cell activation. (*A*) Overview of the experimental approach. CD3^+^ and CD8^+^ T cells were isolated by negative selection (see Materials and Methods) and T cells of each healthy donor were cultivated in the presence of interleukin (IL) 2. CD3^+^ T cells were additionally activated with anti-CD3/28. Cells were stimulated with S100A8, S100A9, and S100A8/S100A9 (1:1), while vehicle served as a control. Surface markers were analyzed after 24 hours, and intracellular cytokine staining was performed after 48 hours of stimulation by flow cytometry. Results are normalized to vehicle and depicted as percentage change relative to control. Effector memory T_EM_ were defined as CD62L^−^/CD45RO^+^, and CD25 and CD69 were used as activation markers. CD8^+^ T cells were additionally analyzed by bulk RNA sequencing and Western blot. FACS, fluorescence-activated cell sorter. Image was created in https://BioRender.com. (*B* and *C*) Quantification of activated CD4^+^ T cells after stimulation with S100A8, S100A9, and S100A8/S100A9 (1:1) for 24 hours (n = 6/6/6/6; Kruskal-Wallis test with Dunn’s correction; median shown). (*D*) Quantification of effector memory CD4^+^ T cells after stimulation with S100A8, S100A9, and S100A8/A9 for 24 hours (n = 6/6/6/6; Kruskal-Wallis test with Dunn’s correction; median shown). (*E* and *F*) Quantification of activated CD8^+^ T cells after stimulation with S100A8, S100A9, and S100A8/S100A9 (1:1) for 24 hours (n = 6/6/6/6; Kruskal-Wallis test with Dunn’s correction; median shown). (*G*) Quantification of effector memory CD8^+^ T cells after stimulation with S100A8, S100A9, and S100A8/S100A9 (1:1) for 24 h (n = 6/6/6/6; Kruskal-Wallis test with Dunn’s correction; median shown). (*H*) Quantification of intracellular granzyme B (GrzB) in CD8^+^ T cells after stimulation with S100A8, S100A9, and S100A8/S100A9 (1:1) for 48 h (n = 6/6/6/6; Kruskal-Wallis test with Dunn’s correction; median shown). (*I*) Quantification of secreted IL17A in the supernatant of CD3^+^ T cells after stimulation with S100A8, S100A9, and S100A8/S100A9 (1:1) for 48 hours (n = 6/6/6/6; Kruskal-Wallis test with Dunn’s correction; median shown). (*J*) Heat map showing activity (act) scores (obtained with a multivariate linear model) of pathways in CD8^+^ T cells obtained from healthy individuals treated with vehicle, S100A8, S100A9, or S100A8/S100A9 (1:1). The *y*-axis indicates treatment condition, the *x*-axis indicates regulated pathways. Red indicates up-regulation of a pathway relative to vehicle, blue indicates down-regulation. Representative immunoblot of CD8^+^ T cells after S100A8, S100A9 and S100A8/S100A9 (1:1) stimulation for the (*K*) nuclear factor (NF)-*κ*B pathway (n = 6) and (*L*) signal transducer and activator of transcription 3 (STAT3) (n = 7). EGFR, epidermal growth factor receptor; GAPDH, glyceraldehyde-3-phosphate dehydrogenase; IKK, I*κ*B kinase; JAK/STAT, Janus kinase/signal transducer and activator of transcription; MAPK, mitogen-activated protein kinase; NF*κ*B, nuclear factor-*κ*B; p-, phosphorylated; PI3K, phosphatidylinositol-3-kinase; TGF, transforming growth factor; TNF, tumor necrosis factor; TRAIl, TNF-related apoptosis-inducing ligand; VEGF, vascular endothelial growth factor. *P < .05, **P < .01, ***P < .001.

Oral exposure to human S100A9 homodimers promoted the recruitment of CD8^+^ T cells into the mucosa of *S100a9*^−/−^ mice (Supplementary Figure 4A–L), without histologic signs of gut inflammation (Supplementary Figure 5A and B). For this reason, we explored the impact of S100A8 and S100A9 homodimers on CD4^+^ and CD8^+^ T cells that are critically involved in chronic gut inflammation in IBD.^24^ We purified CD4^+^ and CD8^+^ T cells from the blood of 6 healthy donors and exposed these cells to S100A8 or S100A9 homodimers, CP, or vehicle, followed by immunophenotyping by flow cytometry (Figure 5A). S100A8 and S100A9 homodimers promoted the activation of CD4^+^ and CD8^+^ effector T cells and effector memory CD45RO^+^ T cells, by enhancing CD25 and CD69 up-regulation upon Tcell receptor stimulation compared with vehicle (Figure 5B–*G* and Supplementary Figure 6B–I). Moreover, S100A9 homodimers increased granzyme B-expressing CD8^+^ T cells (Figure 5H), and S100A8 and S100A9 homodimers induced the production of interleukin 17A, but not interferon gamma or tumor necrosis factor, in CD3^+^ T cells (Figure 5I and Supplementary Figure 6J and K). Furthermore, S100A8 and S100A9 homodimers induced a transcriptional nuclear factor-*κ*B and Janus kinase/signal transducer and activator of transcription signature compared with vehicle, as assessed by bulk RNA sequencing of CD8^+^ T cells (Figure 5J), which we confirmed by immunoblotting (Figure 5K and L and Supplementary Figure 6L–N).

We further analyzed antigen-specific CD4^+^ T-cell activation by S100A8 and S100A9 using antigen-reactive CD4^+^ T-cell enrichment.^25,26^ We detected reactive memory CD4^+^ T cells against S100A9 homodimers, as compared to vehicle (Supplementary Figure 6O). Collectively, these studies demonstrated that human S100A8 and S100A9 homodimers enhance the activation of CD4^+^ and CD8^+^ T cells.

### Inflammatory Actions of S100A8 and S100A9 Homodimers Require Adaptive Immunity in Mice

**Figure 6.**
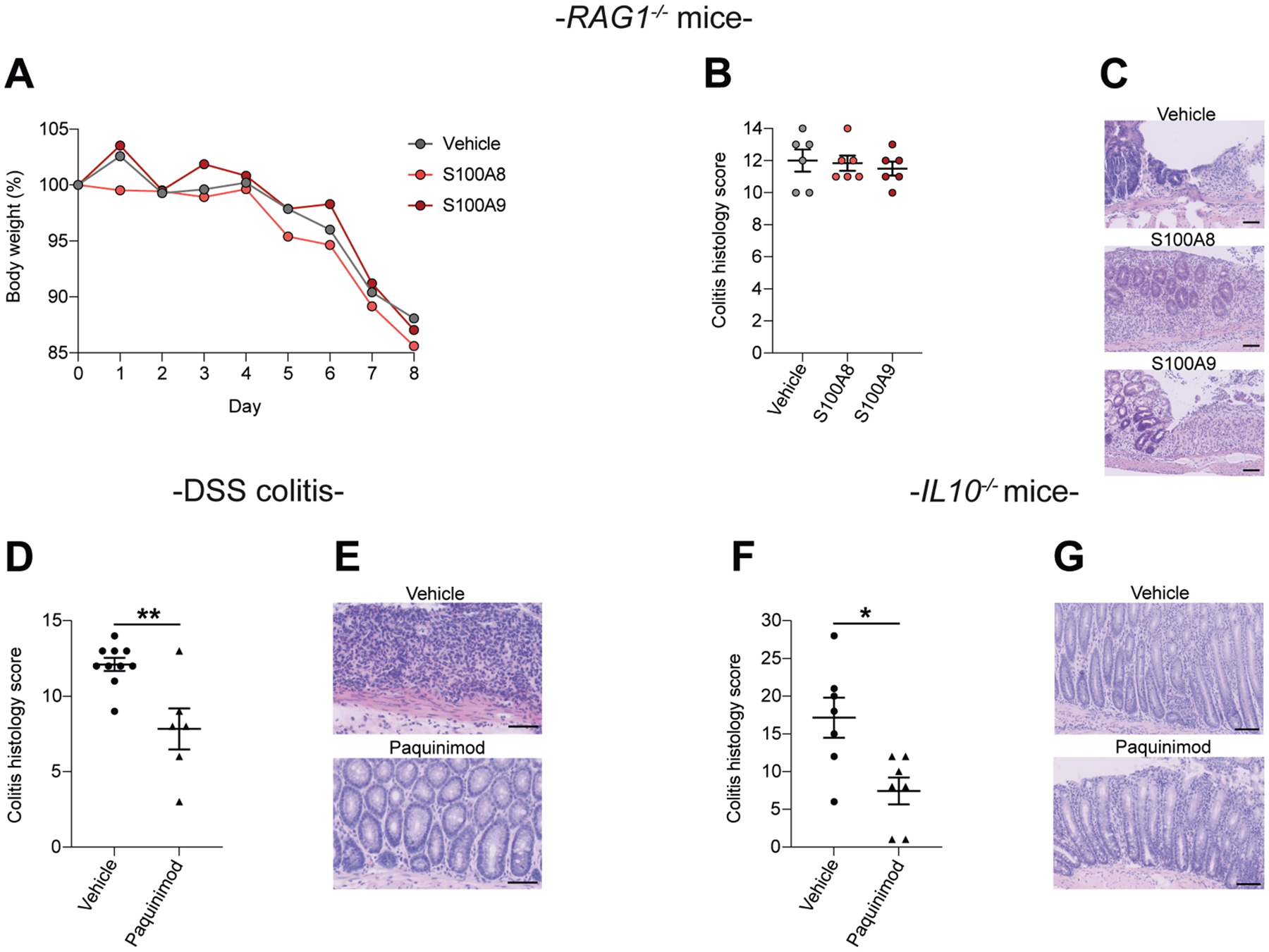
The colitogenic effect of human S100A8 and S100A9 homodimers requires adaptive immunity. (*A*) Body weight course, (*B*) colitis histology score, and (*C*) representative H&E images of *Rag1*^−/−^ mice exposed to DSS and oral gavage with vehicle, S100A8, or S100A9 (n = 6/6/6; 8–9 weeks; mean ± standard error of the mean [SEM] shown; 1-way analysis of variance [ANOVA] with post hoc Bonferroni). *Scale bars*, 50 *μ*m. (*D*) Colitis histology score and (*E*) representative H&E images of WT mice exposed to DSS and treated with paquinimod or vehicle (n = 10/6; 8–9 weeks; mean ± SEM shown; two-tailed Student’s *t*-test). Note the shared vehicle group with experiment shown in in Figure 3A–D, as experiments were performed simultaneously. *Scale bars*, 100 *μ*m. (*F*) Colitis histology score and (*G*) representative H&E images of *Il10*^−/−^ mice treated with paquinimod or vehicle (n = 7/7; 7–8 weeks; mean ± SEM shown; two-tailed Student’s *t*-test). *Scale bars*, 100 *μ*m. *P < .05, **P < .01.

Finally, we validated that human S100A8 and S100A9 homodimers promote intestinal inflammation by induction of adaptive immunity. We induced colitis by DSS in *Rag1*^−/−^ mice that lack adaptive immunity and orally exposed mice to human S100A8 or S100A9 homodimers or vehicle for 4 consecutive days. The inflammatory effect of S100A8 and S100A9 homodimers was comparable to that of vehicle in *Rag1*^−/−^ mice during DSS colitis (Figure 6A–C). These findings demonstrate that proinflammatory actions of S100A8 and S100A9 homodimers were dependent on adaptive immunity, which orchestrate chronic gut inflammation.^27^ Moreover, pharmacologic inhibition of S100A9 with paquinimod (Supplementary Figure 7A) protected WT mice against DSS colitis (Figure 6D and E), and *Il10*^−/−^ mice against chronic colitis (Figure 6F and G). Collectively, our approach demonstrates that human S100A8 and S100A9 homodimers promote experimental colitis by inducing adaptive immune responses and that chronic colitis can be ameliorated by pharmacologic inhibition of S100A9.

## Discussion

IBDs comprise a spectrum of chronic inflammatory disorders with heterogeneous clinical manifestations and a variable response to medical therapy.^3,4^ Despite this complexity, most patients with active IBD exhibit CP in stool, with a fecal CP concentration >150 *μ*g/g being compatible with active disease in symptomatic patients.^5,6^ Understanding the configuration and related biological actions of S100A8 and S100A9 in the human intestine harbors the potential for biomarker discovery in inflammatory conditions of the intestine and may enable therapeutic targeting of a molecular driver of intestinal inflammation in IBD.

Here, we reveal the presence of abundant S100A8 and S100A9 homo-/heterodimers in addition to CP in stool from adults with IBD by size-exclusion chromatography coupled with LC-MS/MS. Tissue transcriptomics from the IBDome cohort^17^ indicated that *S100A8* and *S100A9* were concomitantly expressed in the inflamed mucosa of patients with IBD and that their expression correlated with clinical, endoscopic, and histologic disease activity. By fecal analysis of IBD patients from 2 independent cohorts in Groningen and Innsbruck, we report that S100A8 dimers were detected in 48% and S100A9 dimers were detected in 15% of IBD patients. Fecal S100A9 detection, but not S100A8 detection, provided odds for clinical disease activity in patients with low fecal CP concentration (≤150 *μ*g/g). In an independent cohort from Kiel, Germany, we demonstrate that luminal S100A9 detection associated with clinical and endoscopic disease activity in colonic aspirates from patients with IBD.

Our preclinical studies demonstrate that oral exposure to human S100A8 and S100A9 homodimers, but not CP, worsened gut inflammation in 4 independent mouse models to a comparable extent. In turn, genetic inactivation of *S100a9* protected against experimental gut inflammation in mice. Transcriptional phenotyping of human colonic epithelium and T cells, coupled with mechanistic studies in transgenic mice, portray a mechanism by which S100A8 and S100A9 homodimers promote chronicity of inflammation.

We further focused on enhanced CD4^+^ and CD8^+^ T-cell activity, because these pathways have been implicated in the control of chronic gut inflammation in IBD^27^ and because S100A8 and S100A9 promoted CD4^+^ and CD8^+^ T-cell accumulation during colitis. As such, our study complements previous studies on S100A8 and S100A9 actions during colitis^28,29^ and beyond the intestine, for example in inflammatory liver disorders,^30^ inflammatory skin disease, and autoimmunity.^12–15,31^

Our study further expands the knowledge of S100A8 and S100A9 actions in the human intestine of newborns^9^ and may explain how S100A8 and S100A9 shape intestinal immune maturation, as recently studied in malnourished mice.^32^ We acknowledge that pleiotropic mechanisms may be involved in the immunologic effects of homodimers, for example, previously described Toll-like receptor 4 or receptor for advanced glycation end products activation,^13,14^ which we did not disentangle in our study.

Our study conveys important clinical implications. First, human S100A8 and S100A9 homodimers are not detectable by current clinical fecal CP assays, suggesting a diagnostic gap that could obscure the diagnosis of active IBD in symptomatic patients with low fecal CP (<150 *μ*g/g).

Second, our studies indicate that S100A8 and S100A9 homodimers promote enteritis and colitis in mice, partly by activation of T cells, suggesting that blockade of S100A8 or S100A9 configurations could be used as a strategy to treat IBD.

However, the diagnostic use and therapeutic targeting of S100A8 and S100A9 dimers is limited by several unresolved issues, warranting further investigations. First, our cross-sectional cohorts displayed phenotypic heterogeneity and endoscopy readouts were not available for all cohorts.

Second, the odds to detect clinical disease activity with S100A9 in the discovery cohort was corroborated in a relatively small independent cohort (N = 84), which, however, also indicated an association of S100A9 detection with endoscopic disease activity.

Third, we were unable to identify a clinical phenotype explaining the presence or absence of fecal S100A8 or S100A9 dimers.

To overcome these limitations, large controlled biomarker trials with endoscopy readouts are necessary, which may add clinical value to fecal S100A8 or S100A9 detection in IBD in the future. For instance, recent studies indicated that fecal CP quantification is associated with the probability of clinical, endoscopic, and histologic remission in UC,^33^ and a link with heterogenous CD courses has been identified.^34^

Collectively, our study is notable because it challenges our perception of remission in IBD and specifically the interpretation of low fecal CP concentration in symptomatic IBD patients. Large fecal biomarker trials with endoscopic validation of IBD activity are warranted to determine advantages and disadvantages of fecal S100A9 (or possibly S100A8) dimer detection compared with CP quantification, to guide the use in clinical practice in the future. Beyond the intestine, the clinical utility of S100A9 detection has been suggested by 2 recent studies using patient sera, which indicated that S100A9 and CP could be a specific biomarker for psoriasis arthritis^16^ and that S100A9 could serve as a biomarker for brain metastasis response to radiotherapy.^35^

Our study provides a new perspective for CP subunits as biomarkers for intestinal inflammation in IBD and possibly other inflammatory conditions of the human intestine. Our experimental findings may pave the way for gut-selective medical therapy targeting S100A9 dimers, which can potentially be tailored to patients with IBD by fecal profiling of CP configurations.

## Supplementary Material

Supplementary Information

Note: To access the supplementary material accompanying this article, visit the online version of *Gastroenterology* at www.gastrojournal.org, and at https://doi.org/10.1053/j.gastro.2025.08.040.

## Data Availability

Data sets obtained from single-cell RNA sequencing as well as bulk RNA sequencing are available under https://doi.org/10.5281/zenodo.15222096. The mass spectrometry proteomics data have been deposited to the ProteomeXchange Consortium via the PRoteomics IDEntifications Database (PRIDE) partner repository with the data set identifier PXD068250. Data from the IBDome cohort are publicly available at https://ibdome.org.

## WHAT YOU NEED TO KNOW

### BACKGROUND AND CONTEXT

Fecal calprotectin serves as an inflammatory biomarker in patients with inflammatory bowel disease and is composed of a heterotetrameric protein complex of S100A8 and S100A9 dimers. The quaternary structure (protein configuration) of S100A8 and S100A9 and related biological functions are unknown in the intestine.

### NEW FINDINGS

We reveal the presence of S100A8 and S100A9 dimers, besides calprotectin, in stool from patients with active inflammatory bowel disease and identify human S100A8 and S100A9 homodimers, but not calprotectin, as drivers of enteritis and colitis in mice.

### LIMITATIONS

Large controlled trials with endoscopy endpoints are warranted to establish diagnostic or therapeutic value in targeting S100A8 and S100A9 in inflammatory bowel disease.

### CLINICAL RESEARCH RELEVANCE

Our study opens up new perspectives for S100A8 and S100A9 dimer detection as biomarkers of inflammatory diseases in the intestine. Pharmacologic targeting of S100A8 or S100A9 dimers may be exploited to treat inflammatory bowel diseases in the future.

### BASIC RESEARCH RELEVANCE

Human S100A8 and S100A9 homodimers, but not calprotectin, promote chronicity of intestinal inflammation by pleiotropic mechanisms. These insights can be leveraged to treat inflammatory disorders of the intestine.

## Abbreviations used in this paper:

CD#: cluster of differentiation
CD: Crohn’s disease
CP: calprotectin
DSS: dextran sodium sulfate
ELISA: enzyme-linked immunosorbent assay
GPX4: glutathione peroxidase 4
IBD: inflammatory bowel disease
IEC: intestinal epithelial cells
LC: liquid chromatography
MS/MS: tandem mass spectrometry
UC: ulcerative colitis
WT: wild-type
XBP1: X-box binding protein 1
