# Supplementary material for: Fecal Detection of Calprotectin Subunits Links Inflammatory Bowel Disease Activity With Chronicity of Intestinal Inflammation
Source: Gastroenterology. Author manuscript; Available in PMC 2026 Mar 6. (PMC12964555; doi:10.1053/j.gastro.2025.08.040)
Supplement: Supplementary Information [file NIHMS2139786-supplement-Supplementary_Information.pdf]

Supplementary Information

Supplemental Materials and Methods

S1008 and S100A9 homodimer quantification in stool

Stool samples were diluted 1:10 (w/v) in ice-cold PBS containing EDTA-free protease and phosphatase inhibitors (Thermo Fisher Scientific, 78443). Specimens were vortexed for 30 sec and incubated on a tube rotator at room temperature for 25 min. Homogenates were vortexed for 30 sec and then centrifuged at 10,000 rpm for 20 min. Supernatants were collected and centrifuged for 15 min at 15,000 g and 4°C. Supernatant was stored at -80°C until analysis by ELISA. The following ELISAs were used for human fecal studies: S100A8 (R&D, DY4570-05), S100A9 (R&D, DY5578), S100A8/S100A9 (R&D, DY8226-05), S100A8/A9 (Eurospital Diagnostic, 9031). The following clinically established calprotectin assays were tested for their specificity to detect S100A8 or S100A9: S100A8/A9 (BÜHLMANN, K181012); S100A8/A9 (Euroimmun, EQ 6831-9601 W); S100A8/A9 (Eurospital Diagnostic, 9031); S100A8/A9 (Eurospital Diagnostic, 9300).

S100A8 and S100A9 protein interactors in human stool

Co-immunoprecipitation was performed to identify protein–protein interactions by using S100A8- and S100A9-specific antibodies to indirectly capture interaction partners of both target proteins. Per sample 250 µg of Pierce Magnetic Beads (Thermo Fisher Scientific, 88816) were transferred to a 1.5 mL low protein binding tube (Thermo Scientific, 90410) and separated on a magnetic rack. Beads were washed with PBS containing 0.1% CHAPS (ROTH, 1479.1) at pH 7.4 by shaking the tube thoroughly for 30 sec. Washing was repeated five times. Beads were then incubated with 6 µg of S100A8 (abcam, ab92331) or S100A9 (cell signaling, 72590S) antibody dissolved in 1 mL of incubation buffer (PBS containing 0.1% CHAPS, 0.1% Tween and 1% BSA at pH 7.4) on a rotator at 40 rpm and RT for 2 h. Samples were then washed five times to remove any remains of unconjugated antibodies. Washed beads were incubated with 80 µl of stool supernatant or vehicle diluted in 920 µl of binding buffer (PBS containing 0.1% CHAPS and 0.2 pmol protease inhibitors at pH 7.4) on a rotator at 40 rpm and RT for 1 h. Samples were then incubated overnight on a rotator at 40 rpm at 4°C. Beads were washed five times, resuspended in 200 µL of 1% formic acid (Fisher Chemical, A117-05AMP), vortexed and

incubated at RT for 5 min. Samples were separated on a magnetic rack, supernatants were collected, lyophilized and dissolved in 100 µl twice-distilled water for LC-MS/MS analysis.

Expression and purification of human recombinant S100A8 and S100A9

Plasmid assembly of human recombinant S100A8 and S100A9: Human S100A9 (AA 1-114) and human S100A8 (AA 1-93) were ordered as gBlocks from idtDNA and integrated into the linearized bacterial expression vector pET28a (Novagen, 69866-3) at equimolar concentrations by gibson assembly (NEBuilder® HiFi DNA Assembly Master Mix, E2621S). Expression in the pET28a system is controlled by a T7 promoter that is active only when the plasmid is paired with an appropriate bacterial host that can express the viral T7 polymerase. The pET28a-hS100A9 plasmid and the pET28a-hS100A8 plasmid were separately introduced into chemically competent KRX cells (Promega, L3002) that express T7 polymerase controlled by a rhamnose inducible promoter (rhaBAD). In contrast to recombinant hS100A9 protein, recombinant hS100A8 was predominantly found in insoluble inclusion bodies and remaining hS100A8 was prone to aggregation. This was counteracted by inserting a short (7.6 kDA) N' terminal fusion tag (FH8) to S100A8, which enhanced solubility and stability. Purification was improved by introducing an additional 6xHIS tag preceding FH8. Furthermore, a TEV cleavage site was introduced between fusion tag and hS100A8 to enable protein cleavage. The optimized gBlock was induced in the linearized bacterial expression vector pET28a and resulted in the final pET28a-6xHIS-FH8-hS100A8 (AA 2-93) expression plasmid.

Expression: Transformed bacteria were transferred into terrific broth containing 50 µg mL-1 kanamycin and incubated on a rotation incubator at 250 rpm and 37°C O/N. Overnight cultures (10 mL) were diluted 1:20 in terrific broth containing 50 µg mL-1 kanamycin and incubated on a rotation incubator at 250 rpm and 37°C until an OD600 of 1 was reached. Protein expression was induced by supplementing the medium with 1 mL of 20% rhamnose (w/v) and 200 µl 1M IPTG. Cultures were incubated at 250 rpm and 25°C for 25 h and bacteria were harvested via centrifugation at 6000 g and 4°C for 10 min. The supernatants were discarded and cell pellets were stored at -20°C until further preparation. Cell lysis and extraction: Bacteria were lysed according to manufacturer's instructions (NZYtech, MB17802). Briefly, cells were resuspended in 5 mL of NZY Bacterial Cell Lysis Buffer

(containing 10 mg mL-1 lysozyme, 250 mg mL-1 DNase I and 1x EDTA-free protease inhibitors) per gram cell paste and incubated on a tube rotator for 25 min at RT. Proteins were harvested through centrifugation for 15 min at 15,000 g and 4°C. The collected supernatants contained recombinant human S100A8 and S100A9. Purification of recombinant human S100A9: S100A9 protein was purified through anion-exchange chromatography using an ÄKTA purification system. The sample was diluted to 500 mL with buffer consisting of 50 mM TRIS at pH 7.5, 10 mM NaCl, 1g L-1 colistin and 2.5 mM beta-mercaptoethanol and loaded at 5 mL min−1 onto an equilibrated 5 mL HiTrap CaptoQ ImpRes column (Cytiva, 17547055). Unbound proteins were washed from the column using AIEX Running Buffer (50 mM Tris pH 7.5 at 25°C, 50 mM NaCl and 0.001% Pluronic F68). Elution of recombinant S100A9 was performed with AIEX Elution Buffer (5 mM Tris pH 7.5 at 25°C, 1 M NaCl and 0.001% Pluronic F68) at a concentration of 30% with 10CV at a flow rate of 1 mL min−1. Elution fractions of 1 mL were collected in a 96-well master block and analysed. Fractions specific for human S100A9 were collected, pooled and purified using immobilized metal ion affinity chromatography (IMAC). For this purpose, two columns of 1 mL HisTrap excel were run in tandem to achieve the necessary protein binding capacity. The sample was diluted 1:2 with IMAC Running Buffer (25 mM Hepes pH 7.5 at 25°C, 0.5 M NaCl, 5 mM imidazole and 0.001% Pluronic F68) and loaded onto the equilibrated columns. After a 5CV washing step with running buffer, S100A9 was eluted with 5% IMAC Elution Buffer (1 M imidazole pH 7.5 at 25°C, 0.5 M NaCl and 0.001% Pluronic F68) in 15CV.

Purification of recombinant S100A8 protein: S100A8 protein was purified based on its His-FH8 tag using immobilized metal ion affinity chromatography. The sample was diluted to 250 mL in buffer containing 50 mM Hepes pH 7.5, 0.4 M NaCl, 2.5 mM imidazole and 1 g L-1 colistin. The solution was loaded at 2 mL min−1 onto 1 mL HisTrap excel columns (Cytiva, 17371205) run in tandem. Unbound proteins were washed first with 7.5 CV IMAC Running Buffer and second with 5 CV 2% IMAC Elution Buffer. Elution was performed using 15% elution buffer at a flow rate of 1 mL min−1 with 15CV. Elution fractions of 1 mL were collected and analyzed. TEV cleavage: TEV protease was incubated with FH8-S100A8 protein at a concentration of 1U µg-1 protein for 48 h at 4°C. The cleaved S100A8 was recovered by absorption of the FH8 tag and the TEV protease onto PureCube Ni-INDIGO MagBeads (Cube Biotech, 75205). The remaining supernatant contained the cleaved S100A8 protein.

Quality testing and protein storage: Purity of proteins was assessed with Coomassie gel and LC-MS/MS analysis. Protein constitution was validated through size-exclusion chromatography. After quality testing, proteins were concentrated and dissolved in ice-cold PBS using a 10 kDa molecular weight cut-off spin filter. Samples were concentrated a minimum of five times at 3000 g and 4°C. Proteins were sterile filtered through a 0.22 µm membrane and concentrations were determined using Protein Assay (Bio-Rad Laboratories, 5000006). Recombinant proteins were stored in PBS containing 0.1 % BSA at a concentration of 0.5 µg µl-1 at –80°C. Endotoxin levels within recombinant human S100A8 and S100A9 were assessed using the Pierce LAL Chromogenic Endotoxin Quantitation Kit. Testing was performed according to the manufacturer's instructions (Thermo Scientific, A39552).

Size-exclusion chromatography

Recombinant proteins: For the assessment of complex formation and hydrodynamic radius of the purified proteins we used the ÄktaGO system and a Superdex75 10/300 GL increase column (Cytiva, 29148721) calibrated with the LMW Calibration Kit (Cytiva, 28403841).

Purified S100A9 and/or S100A8 proteins were diluted in running buffer (150 mM NaCl, 25 mM Hepes pH 7.5 at 25°C, 0.05% Pluronic F68) and loaded in a 100 µl injection loop according to manufacturer's specifications (Cytiva). To counteract the propensity of S100 proteins to aggregate during chromatographic separation, 0.75 M guanidium chloride (Sigma, G3272-100G) was supplemented in the injected volume. Heterotetramer and heterodimer formation of S100A8 and S100A9 proteins was achieved with a preceding 1 h pre-incubation in the presence of 10 mM $CaCl_2$.

Stool samples: Fecal samples were prepared as previously described. Apart from the utilized buffers, the procedure was identical to separation of the recombinant S100 proteins. Calibration with the LMW showed a negligible difference between the two buffer systems. PBS was used as running buffer.

Liquid chromatography and tandem mass spectrometry

Sample preparation: Samples in formic acid were lyophilized and re-dissolved in 45 µl of ABC buffer (100mM ammonium bicarbonate, pH 8.0). Proteins were reduced with 5 µl of dithiothreitol (100mM in ABC buffer) at 56°C for 30 min, digested with 0.5 µg of trypsin (Promega, V5111) for 6 h at 37°C, and

alkylated with 50 µl of iodoacetamide (55mM in ABC buffer) for 20 min at RT. Resulting peptides were lyophilized to a volume of 10 µl and stored at -20°C until analysis. Liquid chromatography coupled to mass spectrometry (nanoLC-MS/MS): Peptide digests were analyzed using an UltiMate 3000 nano-HPLC system coupled to a Q Exactive Plus Mass Spectrometer (Thermo Scientific) as described previously. Peptides were separated on a 17 cm long column (100 µm i.d.) packed with 2.4 µm C18 material (Reprosil). Solvents for nano-HPLC were 0.1% formic acid and 0.1% formic acid in 85% acetonitrile. Total gradient time was 82 min at a flow rate of 300 nL min-1. The 20 most abundant peptides in the full MS scan were selected for MS fragmentation. Isolation window was set to 1.6 *m/z*. Full scan spectra were acquired from 300 to 1750 *m/z* at a resolution of 60 000. Peptides were fragmented by HCD with a normalized collision energy set to 28 and scanned at a resolution of 30 000. Database search: The MS data files were processed using Proteome Discoverer, version 2.2 (Thermo Scientific) in combination with the Sequest HT search engine. MS/MS spectra were searched against the Uniprot human reference proteome database with the following search parameters: Enzyme specificity was set to trypsin with two missed cleavages being allowed. Variable modifications were carbamidomethyl on cysteine, oxidation of methionine and acetylation and/or methionine loss of protein N-termini. Precursor mass tolerance was set to 10 ppm; fragment mass tolerance was 20 mmu. Maximum false discovery rate (FDR) for protein and peptide identification was set to 1%. For label-free quantification the Minora Feature Detector node was set to high confidence PSM (peptide spectrum matches) only with at least two isotopic peaks present in the isotope pattern. Retention time alignment was performed at a maximum retention time shift of 10 min and a mass tolerance of 10 ppm. For hypothesis testing, p values were calculated by applying ANOVA (background-based) with Tukey HSD post hoc analysis.

Quantification of S100A8 and S100A9 by LC-MS/MS

For absolute quantification of S100A8 and S100A9, samples were digested equally as described above. Resulting peptides were desalted using Pierce™ C18 pipet tips (Thermo Scientific, 87784), lyophilized and re-solubilized in 32 µl of 0.1% formic acid. To each sample 8 µl of two heavy lysine- (13C15N) labeled standard peptides (Thermo Scientific) was added to obtain final concentrations of 0.272 fmol

µl-1 and 0.312 fmol µl-1 for LGHPDTLNQGEF(K) (S100A9) and ALNSIIDVYH(K) (S100A8), respectively. Each sample was quartered into 10 µl aliquots, three of which were analyzed.

The peptides were separated by nanoLC as described above. The gradient profile was: 0–2 min, 4% B; 2–20 min, 4%–50% B; 20–25 min, 50%–100% B, and 25–30 min, 100% B. The flow rate was 250 nL min-1. For MS analysis, an Orbitrap Eclipse Tribrid mass spectrometer (Thermo Scientific) was operated in data-independent and data-dependent acquisition mode in parallel: full scan spectra were acquired at a resolution of 15000 (at m/z = 200) using a scan range of m/z = 375-1500. Automatic gain control target was set to 4e5 with a maximum ionization time of 22 ms. For data-dependent acquisition, low resolution fragment scans of the five most abundant peptides were acquired in the linear ion trap. In parallel data-independent MS/MS acquisition of 17 ions was performed in the orbitrap at a resolution of 15000, normalized target of 200% and maximum ionization time of 22 ms. Isolation window was set to 1.2 Da. Higher-energy collisional dissociation (HCD) was used for both modes of MS/MS acquisition.

Of the 17 ions acquired in data-independent acquisition mode four ions were used for absolute quantification of the two proteins S100-A9 and S100-A8 (endogenous and heavy labeled peptides LGHPDTLNQGEF(K) and ALNSIIDVYH(K), respectively) and another 13 ions were used to evaluate N- or C-terminal protein degradation (Supplementary Table 8).

The MS data files were processed using Skyline 23.1.0.380 (University of Washington). To benchmark the quantitative assay recombinant S100-A8 and S100-A9 were digested and analyzed at concentrations ranging from 53 amol to 10.6 fmol and from 35 amol to 6.9 fmol and on-column, respectively.

Enteritis and colitis assessment

Histology of gut tissue was evaluated on formalin-fixed, paraffin-embedded and hematoxylin- and eosin-stained sections. Disease severity was determined with a semi-quantitative histology scoring by an expert pathologist blinded to sample identity. The histology score for enteritis and colitis is composed of five histological subscores, and was applied as previously reported [1,2].

Immune cell phenotyping

Lamina propria mononuclear cell (LPMC) isolation was initiated by flushing the small intestine multiple times with ice-cold PBS. A 2 cm piece of small intestine was collected, cut into smaller pieces and incubated in IMDM (Gibco, 21056-023) containing 20% FCS, 10 U mL−1 DNAse II (Sigma, D8764) and 128 U mL−1 collagenase (Sigma, C1889) on a shaker at 37°C for 1 h. Cells were passed through a 70 µm cell strainer, washed with PBS and filtered through a 40 µm cell strainer. After an additional wash, cells were dissolved in FACS buffer (PBS containing 2 mM EDTA and 2% FCS) and stained for innate or adaptive immune cell populations, as previously described[1]. Antibodies were diluted 1:200 in FACS buffer and samples were incubated at 4°C for 30 min. Cells were washed and resuspended in FACS buffer. Analysis was performed on a Cytoflex S (Beckman Coulter). DAPI was used as viability stain and singlets were identified by comparing FSC width and FSC area. The gating strategy for the innate immune cell panel is illustrated in Supplementary Figure 4B. All innate immune cells were defined as CD45+, CD3-, CD19-, CD49b-, and DAPI-negative. The gating strategy for the adaptive immune cell panel is depicted in Supplementary Figure 4C. Adaptive immune cells were identified as CD45+, CD11c-, F4/80-, GR1-, and DAPI-negative. Following antibodies were used: MERTK-PE/CY7 (eBioscience, 25-5751-82), CD11b-APC/Cy7 (eBioscience, 47-0112-80), CD45-FITC (eBioscience, 11-0454-82), Ly6G-superbright600 (eBioscience, 63-9668-82), MHCII-AF700 (eBioscience, 56-5321-82), DAPI (Biolegend, 422801), CD3-superbright600 (eBioscience, 63-0031-82), CD4-APC/eFluor 780 (eBioscience, 47-0041-82), CD19-PE/CY7 (Biolegend, 115520), CD8-FITC (Bioscience, 553030), CD11c-eFluor450 (eBioscience, 48-0114-82), F4/80-eFluor450 (eBioscience, 48-4801-82), CD11b-Percp (Biolegend, 101229), NK1.1-PE (Biolegend, 108707), SiglecF (CD170) eF660 (eBioscience, 50-1702-80).

Human T cell phenotyping

Human T cell purification, culture and stimulation: PBMCs were isolated from peripheral blood using Ficoll density gradient centrifugation. Blood was diluted in a ratio of 2:1 in PBS and carefully transferred onto 15 mL Lymphoprep (Serumwerk Bernburg, 1858). Samples were centrifuged with slow acceleration and without breaks for 20 min at 200 g and RT. Blood plasma and thrombocytes were

discarded and samples were centrifuged for 20 min at 460 g and RT. Separated PBMCs were collected, filled with PBS to 50 mL and centrifuged for 15 min at 300 g and RT. Pellet was resuspended, washed with PBS and centrifuged for 10 min at 300 g and RT. Washing was repeated twice and the pellet was appropriately resuspended in PBS. Cell number was determined prior to negative selection of $CD3^+$ or $CD8^+$ T cells according to the user manual (Miltenyi Biotec, 130-096-535, 130-096-495). Briefly, PBMCs were pelleted for 5 min at 300 g and RT, resuspended in MACS buffer containing the respective biotin antibody cocktail and incubated for 5 min at 4°C. Next, MicroBeads were added, and cells were incubated for 10 min at 4°C. Samples were loaded onto a MACS column (Miltenyi Biotec, 130-042-401) and separated in the magnetic field of a MACS Separator (Miltenyi Biotec). Flow-through contained the enriched $CD3^+$ or $CD8^+$ T cell fractions. Cells were counted, resuspended in T cell medium supplemented with 10 ng mL-1 IL-2 (Peprotech, 200-02) and seeded in a density appropriate for downstream experiments. In case of $CD3^+$ T cells (used for flow cytometric and Bio-Plex analyses), stimulation was performed with anti-CD3 (2 µg mL-1 plate bound; BioXcell, BE0001-2) and anti-CD28 (2 µg mL-1 soluble; BioXcell, BE0248) antibodies. FACS analysis: 500,000 $CD3^+$ T cells per well of a 96-well plate were seeded. Medium was supplemented with 10 ng mL-1 of IL-2 (Peprotech, 200-02). Co-stimulation was performed with 2 µg mL-1 of anti-CD28 (BioXcell, BE0248) and 1 µg mL-1 soluble anti-CD3 (BioXcell, BE0001-2). T cells were cultivated in the presence of human S100A8 (10 µg mL-1), human S100A9 (10 µg mL-1), human S100A8/A9 (10 µg mL-1) or vehicle for 24 h at 37°C (surface markers) or for 48 h (cytokines). Samples were harvested and FACS staining was performed. For surface FACS analysis 1/5 of the sample was transferred to a 96-well round bottom plate and supplemented with 160 µl HBSS (PanBiotech, P04-32505) per well. Samples were centrifuged for 2 min at 2000 rpm and 4°C. Supernatants were discarded, cells were incubated in antibody solution at 4°C for 20 min. Cells were washed and resuspended in MACS Buffer (PBS containing 0.5% BSA and 2 mM EDTA).

For intracellular staining, cells were treated with GolgiStop (BD, 554724) and GolgiPlug (BD, 555029) for 4 h; 4/5 of the sample was transferred to a 96-well round bottom plate and centrifuged for 2 min at 2000 rpm and 4°C. Supernatants were collected for Bio-Plex analysis, cells were washed with 100 µl HBSS and centrifuged for 2 min at 2000 rpm and 4°C. Samples were stained with 100 µl of BD

Horizon™ Fixable Viability Stain 780 (BD Horizon, 565388), diluted 1:2000 in HBSS, and incubated in the dark for 10 min at RT. Cells were washed with MACS Buffer, resuspended in 160 µl fixation buffer (Biolegend, 420801), and incubated in the dark for 20 min at RT. Samples were centrifuged and resuspended in 200 µl MACS Buffer for overnight storage in the dark at 4°C. Cells were centrifuged and washed with 160 µl 1x Perm/Wash Buffer (diluted Intracellular Staining Permeabilization Wash Buffer (Biolegend, 421002)). Samples were incubated in antibody solution (Perm/Wash Buffer) in the dark for 20 min at RT. Cells were washed and resuspended in MACS Buffer. Samples were measured with a BD FACSCanto II using the FACS Diva software. Acquired data were analyzed using FlowJo software.

Following antibodies were used: CD25-PerCP-Cyanine 5.5 (Biolegend, 356111); CD45RO-Fluor® 488 (Biolegend, 304212), CD4-Pacific Blue (Biolegend, 317423); CD62L-PE (Biolegend, 304805), CD69-Alexa Fluor® 647 (Biolegend, 310918); CD8a-Brilliant Violet 510 (Biolegend, 301047), granzyme B-FITC (Biolegend, 515403).

Antigen-reactive T cell enrichment (ARTE)

Peripheral Blood Mononuclear Cells (PBMCs) were freshly isolated from EDTA blood by density gradient centrifugation (Biocoll, Biochrom) on the day of blood donation. ARTE was performed as similarly described[3,4]. In brief, 1–2.5 × 10e7 PBMCs were plated in RPMI-1640 medium (Gibco), supplemented with 5% (v/v) human AB serum (Sigma-Aldrich) in 12-well cell culture plates (1-2 x 10e7 PBMCs each well) or 6-well cell culture plates (more than 2 x 10e7 PBMCs per well). PBMCs were stimulated for 7 h with S100A8, S100A9 and S100A8/9 in the presence of 1 µg mL-1 of CD40 pure antibody (Miltenyi Biotec). For the last 2 h, 1 µg mL-1 of Brefeldin A (Sigma-Aldrich) was added. To multiplex the specificities against S100A8, S100A9 and S100A8/9, the differentially stimulated cells were labeled with different concentrations of two CD4 antibody clones (CD4-BV421, BioLegend, clone OKT4, titer 1:20 and 1:200; CD4-APC-Vio770, Miltenyi Biotec, clone M-T466, titers 1:50 and 1:500). For lower concentrations, the respective unconjugated CD4 pure antibody was added at a concentration of 1 µg mL-1 to block intermixing of the barcode label[3]. Barcoded populations were pooled and labeled with CD154-biotin followed by Anti-Biotin MicroBeads (CD154 MicroBead Kit,

Miltenyi Biotec) and magnetically enriched with two sequential MS Columns (Miltenyi Biotec). Staining with fluorochrome-conjugated antibodies was performed on the first column, followed by fixation and intracellular staining on the second column. Frequencies of antigen-specific T cells were determined based on the cell count of CD154^{+} T cells after enrichment, normalized to the total number of CD4^{+} T cells applied on the column. For each stimulation, CD154^{+} background cells enriched from the non-stimulated control were subtracted.

Following antibodies were used: CD4-BV421 (clone: OKT4) (Biolegend), CD4-APC-Vio770 (clone: M-T466) (Miltenyi Biotec), CD154-FITC (clone: REA238), CD45RA-PE-Cy5 (clone: HI100), IFNg-BV785 (clone: 4S.B3).

Cell culture and stimulation

MODE-K, defined as murine immortalized small intestinal epithelial cells (IECs), were kindly provided by D. Kaiserlian. IECs were cultured in high-glucose DMEM (Lonza, BE12-604F) containing 10% FCS (Biochrom, S0115), 10 mM HEPES (Biochrom, L1613), 0.1 mM non-essential amino acids (Gibco, 11140-035), 100 µg mL−1 streptomycin and 100 U mL−1 penicillin (Biochrom, A2213). Human T cells were isolated from healthy volunteers and cultured in RPMI-1640 (PAN Biotech, P04-18500) containing 10% FCS, 0.1 mM non-essential amino acids, 1 mM sodium pyruvate (Sigma, S8636), 5 mM HEPES buffer, 100 µg mL−1 streptomycin, 100 U mL−1 penicillin, 2 mM L-glutamine (Biowest, X0550) and 0.01 % mM beta-mercaptoethanol (Sigma Aldrich, 63689). MODE-K IECs were stimulated with human S100A8 (5 µg mL-1), human S100A9 (5 µg mL-1), human S100A8/A9 (5 µg mL-1) or vehicle for 1 h, 8 h, 12 h or 24 h. T cells were stimulated with human S100A8 (10 µg mL-1), human S100A9 (10 µg mL-1), human S100A8/A9 (10 µg mL-1) or vehicle for 1 h, 4 h, 24 h or 48 h.

Generation and stimulation of human colonic epithelial organoids: Biopsy samples from healthy volunteers were collected during screening colonoscopies after informed consent was obtained. Resected tissues were washed with ice-cold PBS, minced into smaller pieces and incubated in Gentle Cell Dissociation Reagent (Stemcell Technologies, 100-0485) on a rocking platform for 30 min at 4°C. Samples were then centrifuged for 5 min at 290 g and 4°C. Pellets were resuspended in ice-cold DMEM containing 1% BSA and strained through a 70 µm filter. Crypts were counted and seeded within 50 µl

of Matrigel (BD, 356231) on a pre-warmed 24-well plate. Matrigel was solidified at 37°C for 10 min and samples were then incubated with 500 µl IntestiCult Growth Medium (Stemcell Technologies, 06010) containing 100 µg mL−1 streptomycin and 100 U mL−1 penicillin at 37°C with 5% CO2. Medium was changed at an interval of three days and organoids were split in a ratio of 1:6 every seven days. For single-cell RNA sequencing experiments, organoids were cultivated as monolayers according to manufacturer's instructions (Stemcell Technologies). Briefly, organoids were collected, dissolved in 1 mL Gentle Cell Dissociation Reagent and incubated for 10 min at RT. Dissociated organoids were centrifuged for 5 min at 290 g. Pellets were washed with DMEM/F-12 (Gibco, 12634-010) and centrifuged again for 5 min at 290 g. Samples were resuspended in 1 mL of 0.05 % trypsin-EDTA (Gibco, 25300-054) for 10 min at 37°C. Cells were seeded in 96-well plates coated with 5% Matrigel in IntestiCult Growth Medium and incubated with 200 µl IntestiCult™ Organoid Differentiation Medium (Stemcell Technologies, 100-0214) containing 10 µM Y-27632 (dihydrochloride) (Stemcell, 72304), 100 µg mL−1 streptomycin and 100 U mL−1 penicillin. Medium was changed every three days and experiments were performed after seven days as soon as a confluency of >95% was reached. Organoids were stimulated for 6 h at 37°C with 5 µg mL-1 human recombinant S100A8, S100A9 or S100A8/A9.

Cytokine quantification by enzyme-linked immunosorbent assay in culture supernatant

Extracellular proteins were quantified with enzyme-linked immunosorbent assays (ELISA) or Bio-Plex. Supernatants from human T cells (after 48 h of incubation) were collected, centrifuged for 5 min at 300 g and stored at -20°C. Following Bio-Plex Kits were used for human T cells: Bio-Plex Pro Reagent Kit (Bio-Rad, 171304090M), Bio-Plex Pro HuCSP Standards (Bio-Rad, 12007919), Bio-Plex Pro Human Cytokine IL-17A (Bio-Rad, 171B5014M), Bio-Plex Pro Human Cytokine IFN-γ (Bio-Rad, 171B5019M), Bio-Plex Pro Human Cytokine TNF α (Bio-Rad, 171B5026M).

Immunoblot

Proteins were isolated from MODE-K IECs or human $CD8^+$ T cells using M-PER Protein Extraction Reagent (Thermo Fisher Scientific, 78501) containing 1x EDTA and 1x protease and phosphatase

inhibitors (Thermo Fisher Scientific, 78442). Extraction was performed according to manufacturer's instructions and protein quantity was determined by Bradford Assay (Bio-Rad Laboratories, 5000006). Equal amounts of protein were denaturated in Laemmli Buffer for 5 min at 95°C and loaded to SDS-PAGE. Proteins were then transferred to a polyvinylidene fluoride membrane (GE HealthCare, GE10600023). Blots were blocked in 5% skim milk and primary antibody was incubated overnight at 4°C. Proteins were visualized using HRP-conjugated secondary antibodies (CST, 7074) and ECL Select Western Blotting Detection Reagent (Amersham, RPN2235). Densitometry of immunoblots was performed in Image Lab. Following antibodies were used: GAPDH (Cell Signaling, 2118), IKKα (3G12)(Cell Signaling, 11930), IKKβ (D30C6)(Cell Signaling, 8943), NF-κB p65 (Cell Signaling, D14E12) XP® (Cell Signaling, 8242), Phospho-IKKα/β (Ser176/180)(16A6)(Cell Signaling, 2697), Phospho-NF-κB p65 (Ser536)(93H1)(Cell Signaling, 3033), STAT3 (79D7)(Cell Signaling, 4904), Phospho-STAT3 (Tyr705)(D3A7) XP® (Cell Signaling, 9145), S100A8 (abcam, ab92331), S100A9 (Cell Signaling, 72590).

Immunohistochemistry

Immunohistochemistry was performed using standard protocols[1,5].

Immunofluorescence

Sections were deparaffinized using xylene and rehydrated in descending-gradient ethanol. Heat-mediated antigen retrieval was performed in a steamer by incubating the samples in citrate buffer (Vector Laboratories, H-3300) for 20 min. Slides were washed, blocked (Protein Block, serum-free, Dako, X0909) for 30 min at RT and incubated with primary antibody dissolved in REAL Ab Diluent (Dako, S2022) overnight at 4°C. Sections were washed with PBS three times for 5 min and incubated with Alexa Fluor 488 secondary antibody for 1 h at RT. Slides were washed and mounted with Prolong Diamond Antifade Mountant including DAPI staining (Invitrogen, P36962). Imaging was performed with an Axio Observer Z1 confocal microscope (Carl Zeiss) and ImageJ was used for analysis.

Following antibodies were used: S100A8 (1:1000) (abcam, ab92331), S100A9 (1:250) (abcam, ab92507), AlexaFluor 488 Donkey anti-Rabbit IgG (Invitrogen, A21206).

Single-cell RNA Sequencing

Monolayer organoid cultures were washed with pre-warmed PBS and incubated with 200 µl of freshly prepared dissociation reagent (TrypLE™ Express Enzyme (ThermoFisher Scientific, 12604013) mixed in a ratio of 1:1 with Accutase (Sigma Aldrich, A6964) containing DNase II) for 15 min at 37°C. Organoid fragments were resuspended thoroughly and the reaction was halted by adding 200 µl of DMEM/F-12 medium containing 10% FCS (Biochrome, S0115). Cell suspensions were centrifuged for 5 min at 200 g and 4°C. Pellets were resuspended in 600 µl of dissociation reagent and incubated for 6 min at 37°C. Reactions were stopped by adding 600 µl of DMEM/F-12 medium containing 10% FCS and samples were centrifuged for 5 min at 200 g and 4°C. To further break down cell bonds, pellets were resuspended in 600 µl trypsin-EDTA (0.05%) containing DNase II for 2 min at 37°C. Suspensions were mixed with 600 µl of DMEM/F-12 medium containing 10% FCS and centrifuged for 5 min at 200 g and 4°C. Dead cells were removed according to manufacturer's instructions (Miltenyi Biotec, Dead Cell Removal Kit, 130-090-101). Briefly, pellets were dissolved in 100 µl of Micro Beads Solution and incubated for 15 min at RT. Suspensions were then separated over MACS Columns (Miltenyi Biotec) in the magnetic field of a MACS Separator (Miltenyi Biotec). Dead cells were retained in the column, while the viable cell fraction was centrifuged for 5 min at 200 g and 4°C. Pellets were dissolved in 100 µl ice-cold PBS containing 0.04% BSA. Single-cell suspensions were submitted to the Medical University of Innsbruck MultiOmics Sequencing Core facility for quality control, counting, and generation of single-cell transcriptome libraries applying the Chromium Controller and the Chromium Automated Single-Cell 3′ Reagent Kits User Guide (v3.1) chemistry and protocol from 10x Genomics and targeting 8000-10000 cells per sample. The resulting libraries were sequenced with Illumina NovaSeq technology aiming for a minimum of 20.000 read pairs per targeted cell.

Bulk RNA sequencing of CD8$^+$ T cells

After stimulation with human S100A8 (10 µg mL-1), human S100A9 (10 µg mL-1), human S100A8/A9 (10 µg mL-1) or vehicle at 37°C for 4 h, cells were harvested and centrifuged for 5 min at 300 g and 4°C. Medium was discarded and cells were washed once with PBS for 5 min at 300 g and 4°C. RNA isolation was performed according to manufacturer's instructions using an RNeasy Mini Kit (Qiagen, 74104). RNA was immediately stored at -80°C until library preparation. Purified total RNA was submitted to transcriptome analysis at the Medical University of Innsbruck MultiOmics Sequencing Core for the purpose of gene-expression profiling using the QuantSeq 3′ mRNA-Seq Library Prep method (Lexogen, Vienna Biocenter). Quality-validated, barcoded libraries were multiplexed and sequenced using Illumina NovaSeq technology.

Bioinformatics analysis

Single-cell RNA sequencing data analysis: 10x fastq sequencing files were processed with the Cell Ranger v7.1.0 (10x Genomics) using the nf-core scRNA-seq pipeline v2.4.1[6] with the GRCh38 reference genome and GENCODE v44 annotations. The raw count matrices were loaded into AnnData and further processed with scverse tools [7]. Ambient RNA was removed using scAR as implemented in scvi-tools[8]. Quality control was performed on the denoised counts using scanpy[9], retaining cells with (1) >10000 transcripts, (2) >2000 genes, and (3) <35% mitochondrial transcripts. The 1500 most highly variable genes (HVGs) were selected using scanpy's "highly_variable_genes" function with the options flavor = "seurat_v3" and batch_key = "sample". Cell transcriptomes were embedded into a batch-corrected low-dimensional latent space using scVI[8,10], treating each sample as a separate batch. Doublets were identified and removed using SOLO[11] as implemented in scvi-tools[8]. The neighborhood graph and uniform manifold approximation and projection (UMAP) embedding[12] were computed based on the scVI latent space. Cell types were annotated based on unsupervised clustering with the Leiden algorithm[13] and known marker genes[14,15]. We used DESeq2[16] on pseudo-bulk samples for differential gene expression testing, which has been demonstrated to perform well and properly correct for false discoveries[17]. For each cell type and sample we summed up transcript counts for each gene that is expressed in at least 5% of cells using decoupler-py[18]. P values were adjusted for multiple hypothesis

testing with independent hypothesis weighting (IHW)[19]. Pathway activity analysis was performed with PROGENy[20]. The t-statistics were computed using a multivariate linear model (mlm) as implemented in decoupler-py and the p-values were adjusted for false-discovery rate (FDR). As input, we used test statistics from the DESeq2 analysis ("stat" column). Bulk RNA sequencing data analysis: We used the nf-core/rnaseq (version 3.12.0) pipeline to align the raw reads to the human genome (GRCh38) with STAR and to assess the read counts on the gene models from GENCODE, version 43, with Salmon [6,21,22]. We ran the pipeline with the default parameters except for generating the STAR index, where we used the '--sjdbOverhang 200' parameter, and the gene quantification with Salmon, where we set the parameter '--noLengthCorrection', which accounts for the Lexogen 3' QuantSeq RNA sequencing library. Differentially expressed genes between S100A8, S100A9, S100A8/A9 and paired control samples were calculated using DESeq2 (version 1.38.1) using a fold change threshold of 1.5 and a FDR of 0.1 after Independent Hypothesis Weighting (IHW)[16].

For *pathway activity analysis* we used the R decoupleR[18] package and PROGENy[20] and applied a multivariate linear model to infer pathway activity scores from differential gene expression using the 'stat' statistic from DESeq2 results. We plotted the resulting pathway activity scores as sorted (high to low) heatmap and marked significant enrichment scores (p value <.01). The androgen and estrogen pathways, neither of which was significant or relevant in this context, were removed from the plot.

Bulk RNA sequencing analysis of the IBDome cohort

RNA-sequencing samples were processed using the nf-core RNA-seq pipeline (version 3.4)[6]. In brief, sequencing reads were aligned to the human reference genome (hg38/GRCh38) using STAR (version 2.7.7a)[21] with GENCODE v33 annotations. Quantification of read counts and transcripts per million (TPM) was performed using Salmon[22].

Subsequent data analyses were conducted in R (version 4.2.3). Visualization of the results was performed using the ggplot2 (version 3.5.1) packages. Pearson correlation coefficients were calculated and displayed in scatter plots using the ggpubr package (version 0.6.0), with statistical significance determined by a p-adjusted cutoff of 0.05.

Statistical analysis

The proportions of patients with detectable S100A8 and S100A9 are presented together with 95% Clopper-Pearson confidence intervals. To assess the association between detectable S100A8 and S100A9 and active disease, logistic regression models were used without adjustment, adjusted for age and sex, and adjusted for age, sex, and type of disease (CD vs. UC). We took into account that data stemmed from two different cohorts by including cohort as an additional covariate in all models. Data are expressed as mean ± standard error of the mean (SEM), unless otherwise stated. For each experiment sample size (n) is indicated in the figure legend. GraphPad Prism 10 and Stata 15.1 MP was used for statistical analysis. Grubbs test was used for outlier testing. Statistical significance was assessed with an unpaired two-tailed Student's T test, a Mann-Whitney U test, a one-way ANOVA with Bonferroni correction or a Kruskal-Wallis test with Dunn's correction (as appropriate and stated in the figure legends), and was assumed at *$P < .05$, **$P < .01$, ***$P < .001$, **** $P < .0001$.

Supplemental References

1 Schwarzler, J. *et al.* PUFA-Induced Metabolic Enteritis as a Fuel for Crohn's Disease. *Gastroenterology* **162**, 1690-1704 (2022). https://doi.org/10.1053/j.gastro.2022.01.004

2 Adolph, T. E. *et al.* Paneth cells as a site of origin for intestinal inflammation. *Nature* **503**, 272-276 (2013). https://doi.org/10.1038/nature12599

3 Martini, G. R. *et al.* Selection of cross-reactive T cells by commensal and food-derived yeasts drives cytotoxic T(H)1 cell responses in Crohn's disease. *Nat Med* **29**, 2602-2614 (2023). https://doi.org/10.1038/s41591-023-02556-5

4 Bacher, P. *et al.* Human Anti-fungal Th17 Immunity and Pathology Rely on Cross-Reactivity against Candida albicans. *Cell* **176**, 1340-1355 e1315 (2019). https://doi.org/10.1016/j.cell.2019.01.041

5 Mayr, L. *et al.* Dietary lipids fuel GPX4-restricted enteritis resembling Crohn's disease. *Nat Commun* **11**, 1775 (2020). https://doi.org/10.1038/s41467-020-15646-6

6 Ewels, P. A. *et al.* The nf-core framework for community-curated bioinformatics pipelines. *Nat Biotechnol* **38**, 276-278 (2020). https://doi.org/10.1038/s41587-020-0439-x

7 Virshup, I. *et al.* The scverse project provides a computational ecosystem for single-cell omics data analysis. *Nat Biotechnol* **41**, 604-606 (2023). https://doi.org/10.1038/s41587-023-01733-8

8 Gayoso, A. *et al.* A Python library for probabilistic analysis of single-cell omics data. *Nat Biotechnol* **40**, 163-166 (2022). https://doi.org/10.1038/s41587-021-01206-w

9 Wolf, F. A., Angerer, P. & Theis, F. J. SCANPY: large-scale single-cell gene expression data analysis. *Genome Biol* **19**, 15 (2018). https://doi.org/10.1186/s13059-017-1382-0

10 Xu, C. *et al.* Probabilistic harmonization and annotation of single-cell transcriptomics data with deep generative models. *Mol Syst Biol* **17**, e9620 (2021). https://doi.org/10.15252/msb.20209620

11 Bernstein, N. J. *et al.* Solo: Doublet Identification in Single-Cell RNA-Seq via Semi-Supervised Deep Learning. *Cell Syst* **11**, 95-101 e105 (2020). https://doi.org/10.1016/j.cels.2020.05.010

12 Becht, E. *et al.* Dimensionality reduction for visualizing single-cell data using UMAP. *Nat Biotechnol* (2018). https://doi.org/10.1038/nbt.4314

13 Traag, V. A., Waltman, L. & van Eck, N. J. From Louvain to Leiden: guaranteeing well-connected communities. *Sci Rep* **9**, 5233 (2019). https://doi.org/10.1038/s41598-019-41695-z

14 Elmentaite, R. *et al.* Cells of the human intestinal tract mapped across space and time. *Nature* **597**, 250-255 (2021). https://doi.org/10.1038/s41586-021-03852-1

15 Parikh, K. *et al.* Colonic epithelial cell diversity in health and inflammatory bowel disease. *Nature* **567**, 49-55 (2019). https://doi.org/10.1038/s41586-019-0992-y

16 Love, M. I., Huber, W. & Anders, S. Moderated estimation of fold change and dispersion for RNA-seq data with DESeq2. *Genome Biol* **15**, 550 (2014). https://doi.org/10.1186/s13059-014-0550-8

17 Squair, J. W. *et al.* Confronting false discoveries in single-cell differential expression. *Nat Commun* **12**, 5692 (2021). https://doi.org/10.1038/s41467-021-25960-2

18 Badia, I. M. P. *et al.* decoupleR: ensemble of computational methods to infer biological activities from omics data. *Bioinform Adv* **2**, vbac016 (2022). https://doi.org/10.1093/bioadv/vbac016

19 Ignatiadis, N., Klaus, B., Zaugg, J. B. & Huber, W. Data-driven hypothesis weighting increases detection power in genome-scale multiple testing. *Nat Methods* **13**, 577-580 (2016). https://doi.org/10.1038/nmeth.3885

20 Schubert, M. *et al.* Perturbation-response genes reveal signaling footprints in cancer gene expression. *Nat Commun* **9**, 20 (2018). https://doi.org/10.1038/s41467-017-02391-6
21 Dobin, A. *et al.* STAR: ultrafast universal RNA-seq aligner. *Bioinformatics* **29**, 15-21 (2013). https://doi.org/10.1093/bioinformatics/bts635
22 Patro, R., Duggal, G., Love, M. I., Irizarry, R. A. & Kingsford, C. Salmon provides fast and bias-aware quantification of transcript expression. *Nat Methods* **14**, 417-419 (2017). https://doi.org/10.1038/nmeth.4197

Supplementary Tables

Tables

Supplementary Table 1. Characteristics of the IBDome cohort.

Characteristic	CD	UC	non-IBD	total
**No. of samples**	235	125	54	414
Female sex - no. (%)	126 (54)	57 (46)	28 (52)	211 (51)
Age at sampling (years) - mean (SD)	38 (14)	42 (16)	56 (17)	42 (16)
BMI at sampling - mean (SD)	25 (5)	26(5)	-	25 (5)
**disease localization**				
L1: terminal ileum (%)	91 (39)	-	-	-
L2: colon (%)	12 (5)	-	-	-
L3: ileocolon (%)	112 (48)	-	-	-
E1: proctitis (%)	-	7 (6)	-	-
E2: left sided colitis (%)	-	42 (34)	-	-
E3: extensive colitis (%)	-	55 (44)	-	-
**disease activity scores**				
SES-CD - mean (SD)	5.0 (8.9)	-	0 (0)	-
UCEIS - mean (SD)	-	2.1 (2.5)	0 (0)	-
HBI - mean (SD)	3.2 (3.3)	-	-	-
PMS - mean (SD)	-	1.45 (2.38)	-	-
**on treatment***				
Biologics - no. (%)	117 (50)	33 (26)	0 (0)	150 (36)
Conventional therapies - no. (%)	40 (17)	37 (30)	2 (4)	79 (19)
Small molecules - no. (%)	0 (0)	3 (2)	0 (0)	3 (1)
**Characteristic**	**CD**	**UC**	**non-IBD**	**total**
**No. of patients**	152	94	36	282
Female sex - no. (%)	78 (51)	44 (47)	18 (50)	140 (50)
**Smoking (%)**	27 (18)	5 (5)	2 (6)	34 (12)
**previous or ongoing treatment****				
Biologics - no. (%)	108 (71)	47 (50)	0 (0)	155 (55)
Conventional therapies - no. (%)	99 (65)	52 (55)	3 (8)	154 (55)
Small molecules - no. (%)	1 (1)	9 (10)	0 (0)	10 (4)
Probiotics - no. (%)	0 (0)	3 (3)	2 (6)	5 (2)
Non-IBD specific therapies - no. (%)	1 (1)	4 (4)	0 (0)	5 (2)

* Biologics comprise Adalimumab, Golimumab, Infliximab, Ustekinumab, and Vedolizumab; Conventional therapies comprise 5ASA, AZA, Budesonide, Ciclosporin, MTX, Prednisolone, and Sulfasalazine; Small molecules comprise Filgotinib and Tofacitinib;

** Biologics comprise Adalimumab, Golimumab, Infliximab, Risankizumab, Ustekinumab, and Vedolizumab; Conventional therapies comprise 5ASA, 6-MP, AZA, Budesonide, Ciclosporin, Hydrocortisone acetate, MTX, Prednisolone, Sulfasalazine, and Tacrolimus; Small molecules comprise Filgotinib and Tofacitinib; Probiotics comprise E.coli Nissle; and non IBD therapies comprise Ursodeoxycholic acid. Abbreviations: CD, Crohn's disease; UC, Ulcerative colitis; SD, standard deviation; BMI, body mass index; SES-CD, Simple Endoscopic Score for Crohn's Disease; UCEIS, Ulcerative Colitis Endoscopic Index of Severity; HBI, Harvey-Bradshaw Index for Crohn's Disease; PMS, Partial Mayo Score; 5ASA, 5-aminosalicylic acid; AZA, azathioprine; MTX, methotrexate; 6-MP, 6-mercaptopurine.

Supplementary Table 2. Patient characteristics of the IBD cohorts from Innsbruck (Austria) and Groningen (The Netherlands).

Characteristic	Groningen	Innsbruck	Total
Number of patients	316	223	539
Age (years) - mean (SD)	43 (15)	42 (15)	43 (15)
Female sex - no. (%)	188 (59)	113 (51)	301 (56)
CD - no. (%)	184 (58)	154 (69)	338 (63)
Body mass index (kg/m^2) - mean (SD)	25 (5)	24 (4)	25 (5)
Calprotectin detectable - no. (%)			
Patients with CD	130 (71)	141 (92)	271 (80)
Patients with UC	88 (67)	54 (78)	142 (71)
All patients	218 (69)	195 (87)	413 (77)
Calprotectin >150 µg/g - no. (%)			
Patients with CD	36 (20)	68 (44)	104 (31)
Patients with UC	41 (31)	35 (51)	76 (38)
All patients	77 (24)	103 (46)	180 (33)
S100A8 detectable - no. (%)			
Patients with CD	82 (45)	61 (40)	143 (42)
Patients with UC	78 (59)	27 (39)	105 (52)
All patients	160 (51)	88 (39)	248 (46)
S100A9 detectable - no. (%)			
Patients with CD	40 (22)	9 (6)	49 (14)
Patients with UC	31 (23)	3 (4)	34 (17)
All patients	71 (22)	12 (5)	83 (15)
Clinical disease activity - no. (%)*	83 (27)	96 (43)	179 (34)
Calprotectin >150 µg/g	28 (37)	76 (75)	104 (58)
Calprotectin ≤150 µg/g	55 (23)	20 (17)	75 (21)

*Active disease was defined by clinical activity indices available in 534 of 539 patients (99.1%): In the Innsbruck cohort a Crohn's Disease Activity Index >150 among CD patients and a partial Mayo score ≥2 among UC patients indicated clinical disease activity. In the Groningen cohort a Harvey-Bradshaw Index ≥5 among CD patients and an SSCAI score ≥3 among UC patients indicated clinical disease activity. Abbreviations: CD, Crohn´s disease; UC, ulcerative colitis.

Supplementary Table 3. Relation of disease localization with fecal dimer detection.

	Montreal L1*	Montreal L2	Montreal L3	Test	P-value
	**All CD patients**				
	N=79	N=60	N=189		
Fecal calprotectin (µg/g)	53.5 (0.0-179.2)	52.0 (18.3-161.1)	85.7 (25.5-198.2)	Kruskal-Wallis	0.14
Detectable S100A8- no.(%)				Chi-square	0.80
No	43 (54.4%)	35 (58.3%)	111 (58.7%)		
Yes	36 (45.6%)	25 (41.7%)	78 (41.3%)		
Detectable S100A9- no.(%)				Chi-square	0.79
No	69 (87.3%)	53 (88.3%)	161 (85.2%)		
Yes	10 (12.7%)	7 (11.7%)	28 (14.8%)		
	**Patients with calprotectin ≤150 µg/g stool**				
	N=58	N=43	N=128		
Fecal calprotectin (µg/g)	30.9 (0.0-62.9)	29.1 (0.0-56.0)	44.9 (16.1-87.2)	Kruskal-Wallis	0.056
Detectable S100A8- no.(%)				Chi-square	0.85
No	32 (55.2%)	26 (60.5%)	75 (58.6%)		
Yes	26 (44.8%)	17 (39.5%)	53 (41.4%)		
Detectable S100A9- no.(%)				Chi-square	0.72
No	51 (87.9%)	38 (88.4%)	108 (84.4%)		
Yes	7 (12.1%)	5 (11.6%)	20 (15.6%)		

* Montreal L1 is defined as ileal disease, Montreal L2 is defined as colonic disease and Montreal L3 is defined as ileocolonic disease. Abbreviations: CD, Crohn´s disease.

Supplementary Table 4. Relation of smoking status, body mass index, C-reactive protein and hemoglobin concentration with fecal dimer detection.

**Patients with calprotectin ≤150 µg/g stool**				
**S100A8 detectable**	**No**	**Yes**	**Test**	**P-value**
	N=192	N=167		
Smoking status- no. (%)			Chi-square	0.20
Current	40 (21.3%)	27 (16.6%)		
Ex	48 (25.5%)	55 (33.7%)		
Never	100 (53.2%)	81 (49.7%)		
Body mass index	25.0±4.4	24.7±4.5	independent t test	0.56
C-reactive protein (mg/L)	1.10 (0.00-3.50)	1.30 (0.00-5.20)	Wilcoxon rank-sum	0.49
Hemoglobin (g/dL)	14.1±1.6	13.6±1.5	independent t test	0.080
**S100A9 detectable**	**No**	**Yes**	**Test**	**P-value**
	N=303	N=56		
Smoking status- no. (%)			Chi-square	0.014
Current	56 (19.0%)	11 (19.6%)		
Ex	78 (26.4%)	25 (44.6%)		
Never	161 (54.6%)	20 (35.7%)		
Body mass index	24.7±4.3	25.6±5.1	independent t test	0.14
C-reactive protein (mg/L)	1.30 (0.00-3.80)	1.00 (0.00-4.90)	Wilcoxon rank-sum	0.80
Hemoglobin (g/dL)	14.0±1.6	13.3±1.0	independent t test	0.37

Supplementary Table 5. Patient characteristics of the adult IBD cohort from Kiel, Germany.

	Active*		Remission	
Number of patients	65		19	
male/female %	66/34		53/47	
Age ± SD	41.44	17.03	41.28	10.91
BMI ± SD	25.41	5.91	25.20	4.34
CRP (mg/L) ± SD	8.68	14.40	3.14	3.83
Hb (g/dL) ± SD	13.05	2.00	13.44	1.57
CD/UC %	23/77		63/37	
CDAI (CD) ± SD	122.40	82.66	141.58	80.29
Partial Mayo (UC) ± SD	2.98	2.63	1.00	1.07
SES-CD (CD) ± SD	8.07	4.27	0.58	0.76
Endo Mayo (UC) ± SD	1.76	0.76	0	0
Median calprotectin in colonic aspirate (µg/mL)	0.41		0.41	

*Active disease was defined by endoscopic disease activity (SES-CD>2 or Endoscopic Mayo Score >0). Abbreviations: BMI, body mass index; CRP, C-reactive protein; Hb, hemoglobin; CD, Crohn´s disease; UC, ulcerative colitis; CDAI, Crohn´s disease activity index; Partial Mayo, Partial Mayo Scoring Index; SES-CD, Simple Endoscopic Score; Endo Mayo, Endoscopic Mayo Scoring Index.

Supplementary Table 6. Human protein interaction partners of S100A8 in stool from active IBD patients.

S100A8 Co-IP	Abundance Ratio: (S100A8-IP) / (control-IP)	Abundance Ratio P-Value: (S100A8-IP) / (control-IP)	Abundance Ratio Adj. P-Value: (S100A8-IP) / (control-IP)
Matrix metalloproteinase-15 OS=Homo sapiens OX=9606 GN=MMP15 PE=1 SV=1	1000	1.00E-17	3.88E-16
Galectin-4 OS=Homo sapiens OX=9606 GN=LGALS4 PE=1 SV=1	1000	1.00E-17	3.88E-16
ATP synthase subunit beta, mitochondrial OS=Homo sapiens OX=9606 GN=ATP5F1B PE=1 SV=3	1000	1.00E-17	3.88E-16
Disco-interacting protein 2 homolog B OS=Homo sapiens OX=9606 GN=DIP2B PE=1 SV=3	1000	1.00E-17	3.88E-16
Mesencephalic astrocyte-derived neurotrophic factor OS=Homo sapiens OX=9606 GN=MANF PE=1 SV=3	913.72	1.00E-17	3.88E-16
Immunoglobulin heavy constant gamma 4 OS=Homo sapiens OX=9606 GN=IGHG4 PE=1 SV=1	139.92	1.00E-17	3.88E-16
Triple functional domain protein OS=Homo sapiens OX=9606 GN=TRIO PE=1 SV=2	102.96	1.00E-17	3.88E-16
Putative methyltransferase NSUN5C OS=Homo sapiens OX=9606 GN=NSUN5P2 PE=5 SV=2	80.71	1.00E-17	3.88E-16
Immunoglobulin kappa variable 4-1 OS=Homo sapiens OX=9606 GN=IGKV4-1 PE=1 SV=1	48.63	4.88E-15	1.63E-13
A disintegrin and metalloproteinase with thrombospondin motifs 17 OS=Homo sapiens OX=9606 GN=ADAMTS17 PE=2 SV=2	35.41	7.02E-13	2.18E-11
Semaphorin-3A OS=Homo sapiens OX=9606 GN=SEMA3A PE=1 SV=1	32.99	2.01E-12	5.86E-11
Inhibitor of Bruton tyrosine kinase OS=Homo sapiens OX=9606 GN=IBTK PE=1 SV=3	21.51	7.40E-10	1.86E-08
NACHT, LRR and PYD domains-containing protein 1 OS=Homo sapiens OX=9606 GN=NLRP1 PE=1 SV=1	20.32	1.53E-09	3.66E-08
Immunoglobulin heavy variable 3-43 OS=Homo sapiens OX=9606 GN=IGHV3-43 PE=3 SV=1	18.05	6.68E-09	1.45E-07
Protein S100-A9 OS=Homo sapiens OX=9606 GN=S100A9 PE=1 SV=1	15.20	5.13E-08	1.04E-06

Protein S100-A8 OS=Homo sapiens OX=9606 GN=S100A8 PE=1 SV=1	14.20	1.12E-07	2.22E-06
Immunoglobulin kappa variable 1-9 OS=Homo sapiens OX=9606 GN=IGKV1-9 PE=3 SV=1	13.50	1.96E-07	3.72E-06
Chymotrypsin-like elastase family member 2A OS=Homo sapiens OX=9606 GN=CELA2A PE=1 SV=1	11.25	1.37E-06	2.45E-05
Immunoglobulin heavy constant gamma 3 OS=Homo sapiens OX=9606 GN=IGHG3 PE=1 SV=2	11.14	1.51E-06	2.66E-05
DNA primase large subunit OS=Homo sapiens OX=9606 GN=PRIM2 PE=1 SV=2	11.01	1.71E-06	2.95E-05
Creatine kinase U-type, mitochondrial OS=Homo sapiens OX=9606 GN=CKMT1B PE=1 SV=1	10.35	3.19E-06	5.31E-05
ADP-ribosylation factor 3 OS=Homo sapiens OX=9606 GN=ARF3 PE=1 SV=2	8.97	1.27E-05	1.97E-04
Immunoglobulin kappa variable 1D-39 OS=Homo sapiens OX=9606 GN=IGKV1D-39 PE=3 SV=2	7.48	6.48E-05	9.90E-04
Carboxypeptidase A2 OS=Homo sapiens OX=9606 GN=CPA2 PE=1 SV=3	6.62	1.81E-04	2.68E-03
Cofilin-1 OS=Homo sapiens OX=9606 GN=CFL1 PE=1 SV=3	6.22	2.98E-04	4.34E-03
Zymogen granule membrane protein 16 OS=Homo sapiens OX=9606 GN=ZG16 PE=1 SV=2	5.43	8.45E-04	1.09E-02
Protein S100-A12 OS=Homo sapiens OX=9606 GN=S100A12 PE=1 SV=2	5.22	1.13E-03	1.45E-02
Histone H2B type 1-K OS=Homo sapiens OX=9606 GN=HIST1H2BK PE=1 SV=3	4.83	1.95E-03	2.43E-02
Immunoglobulin kappa variable 6D-21 OS=Homo sapiens OX=9606 GN=IGKV6D-21 PE=3 SV=1	4.77	2.14E-03	2.62E-02
Integrin beta-3 OS=Homo sapiens OX=9606 GN=ITGB3 PE=1 SV=2	4.66	2.51E-03	2.92E-02
Alpha-2-macroglobulin OS=Homo sapiens OX=9606 GN=A2M PE=1 SV=3	4.38	3.75E-03	4.11E-02
Immunoglobulin kappa joining 1 OS=Homo sapiens OX=9606 GN=IGKJ1 PE=4 SV=2	4.29	4.32E-03	4.62E-02

Supplementary Table 7. Human protein interaction partners of S100A9 in stool from active IBD patients.

S100A9 Co-IP	Abundance Ratio: (S100A9-IP) / (control-IP)	Abundance Ratio P-Value: (S100A9-IP) / (control-IP)	Abundance Ratio Adj. P-Value: (S100A9-IP) / (control-IP)
Matrix metalloproteinase-15 OS=Homo sapiens OX=9606 GN=MMP15 PE=1 SV=1	1000	1.00E-17	4.66E-16
CD177 antigen OS=Homo sapiens OX=9606 GN=CD177 PE=1 SV=2	585.84	1.00E-17	4.66E-16
ATP synthase subunit beta, mitochondrial OS=Homo sapiens OX=9606 GN=ATP5F1B PE=1 SV=3	559.69	1.00E-17	4.66E-16
Disco-interacting protein 2 homolog B OS=Homo sapiens OX=9606 GN=DIP2B PE=1 SV=3	156.95	1.00E-17	4.66E-16
Galectin-4 OS=Homo sapiens OX=9606 GN=LGALS4 PE=1 SV=1	90.53	1.00E-17	4.66E-16
Immunoglobulin heavy constant gamma 4 OS=Homo sapiens OX=9606 GN=IGHG4 PE=1 SV=1	20.88	1.00E-17	4.66E-16
Triple functional domain protein OS=Homo sapiens OX=9606 GN=TRIO PE=1 SV=2	20.52	1.00E-17	4.66E-16
Immunoglobulin heavy variable 2-26 OS=Homo sapiens OX=9606 GN=IGHV2-26 PE=3 SV=1	7.65	2.14E-10	9.07E-09
NACHT, LRR and PYD domains-containing protein 1 OS=Homo sapiens OX=9606 GN=NLRP1 PE=1 SV=1	6.22	1.33E-08	4.95E-07
Immunoglobulin heavy constant gamma 3 OS=Homo sapiens OX=9606 GN=IGHG3 PE=1 SV=2	5.48	1.37E-07	4.41E-06
Cilia- and flagella-associated protein 74 OS=Homo sapiens OX=9606 GN=CFAP74 PE=2 SV=3	4.43	4.76E-06	1.35E-04
Protein S100-A9 OS=Homo sapiens OX=9606 GN=S100A9 PE=1 SV=1	3.97	2.38E-05	6.33E-04
Protein S100-A8 OS=Homo sapiens OX=9606 GN=S100A8 PE=1 SV=1	3.95	2.56E-05	6.62E-04
DNA primase large subunit OS=Homo sapiens OX=9606 GN=PRIM2 PE=1 SV=2	3.87	3.43E-05	8.65E-04
Immunoglobulin kappa variable 1-9 OS=Homo sapiens OX=9606 GN=IGKV1-9 PE=3 SV=1	3.43	1.81E-04	4.12E-03
Immunoglobulin kappa variable 1D-8 OS=Homo sapiens OX=9606 GN=IGKV1D-8 PE=3 SV=6	3.17	4.76E-04	1.06E-02

Supplementary Table 8. Mass list table used for spike-in experiments.

Protein	Peptide Sequence	Modification	Position in Protein	m/z	z	t start (min)	stop (min)	HCD Collision Energy (%)
S100A8	ALNSIIDV YHK		8-18	424,9031	3	12,88	15,88	30
S100A8	ALNSIIDV YHK	Label 13C15N [K18]	8-18	427,5666	3	12,88	15,88	30
S100A8	GNFHAV YR		24-31	482,2436	2	9,11	12,11	33
S100A8	LLETECP QYIR	Carbamido-methyl [C42]	37-47	711,3583	2	12,56	15,56	30
S100A8	KGADVW FK		49-56	475,7584	2	11,18	14,18	33
S100A8	GADVWF K		50-56	411,7109	2	13,01	14,01	30
S100A9	MSQLER	Oxidation [M5]	5-10	390,1894	2	7,99	10,99	30
S100A9	MSQLER		5-10	382,1918	2	8,78	11,78	30
S100A9	NIETIINT FHQYSVK		11-25	602,9843	3	15,12	18,12	30
S100A9	LGHPDTL NQGEFK		26-38	485,9124	3	10,64	13,64	33
S100A9	LGHPDTL NQGEFK	Label 13C15N [K38]	26-38	488,5786	3	10,64	13,64	33
S100A9	DLQNFLK		44-50	439,2425	2	14,28	17,28	30
S100A9	VIEHIME DLDTNA DK		58-72	581,6137	3	13,02	16,02	30
S100A9	VIEHIME DLDTNA DK	Oxidation [M63]	58-72	586,9453	3	11,37	14,37	30
S100A9	LTWASHE K		86-93	486,2511	2	9,17	12,17	30
S100A9	MHEGDE GPGHHH KPGLGEG TP		94-114	544,7461	4	8,5	11,5	30
S100A9	MHEGDE GPGHHH KPGLGEG TP	Oxidation [M9]	94-114	548,7448	4	8,07	11,07	30

Supplementary Figures

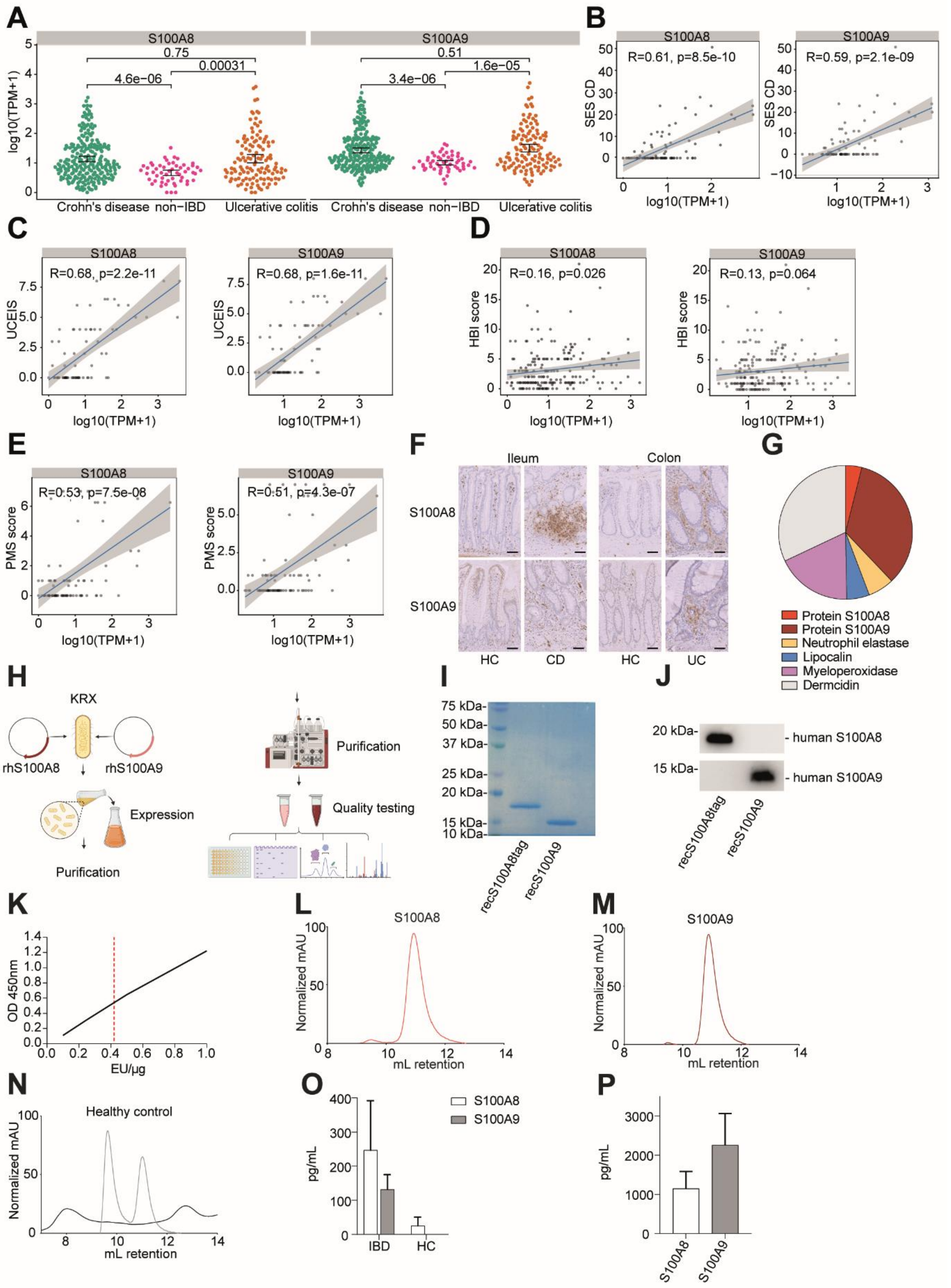

Supplementary Figure 1. Generation of human recombinant S100A8 and S100A9. A. Mucosal expression levels of *S100A8* and *S100A9* in patients with Crohn´s disease (green) and Ulcerative Colitis (orange) compared to non-IBD patients (pink) with P-values retrieved with the Wilcoxon-Mann-

Whitney test. **B, C.** Mucosal expression levels of *S100A8* and *S100A9* in patients with (**B**) CD and (**C**) UC correlated with endoscopic disease activity as assessed by Simple Endoscopic Score (SES-CD) and Ulcerative Colitis Endoscopic Index of Severity (UCEIS). **D, E.** Mucosal expression levels of *S100A8* and *S100A9* in patients with (**D**) CD and (**E**) UC correlated with clinical disease activity as assessed by Harvey-Bradshaw Index (HBI) and Partial Mayo Score (PMS). **F.** Representative immunohistochemistry images of S100A8 and S100A9 in the ileum of CD patients and colon of UC patients compared to healthy controls (HC). Scale bars, 50 µm. **G.** Relative proportion of abundant proteins consistently detectable in stool of all IBD patients (CD, n=8; UC n=8) analyzed with LC-MS/MS. **H.** Schematic workflow for the production and purification of human recombinant S100A8 and S100A9. Image was created in https://BioRender.com. **I.** Representative Coomassie staining of human recombinant S100A8 and S100A9 protein. **J.** Immunoblot of human recombinant S100A8 and S100A9 protein. **K.** Representative endotoxin test for human recombinant S100A8 and S100A9 (dotted line) depicted in EU per µg of recombinant protein. **L, M.** Representative size-exclusion chromatography (SEC) spectra of human recombinant S100A8 (**L**) and S100A9 (**M**) homodimers. **N.** Representative SEC spectra of stool dissolved in PBS from healthy controls (n=4). Grey spectra are chromatographic peaks of human recombinant calprotectin dimers and tetramers, indicating the SEC fractions containing S100A8/S100A9 dimers in human stool. **O.** Quantification of S100A8 and S100A9 concentration in SEC fractions of stool at a size compatible with homo- or heterodimers in IBD patients with a positive ELISA, and healthy controls (HC) (n=8/4). **P.** Quantification of S100A8 and S100A9 concentration in SEC fractions of endoscopy washes at a size compatible with homo- or heterodimers in CD and UC patients by ELISA (n=3/3).

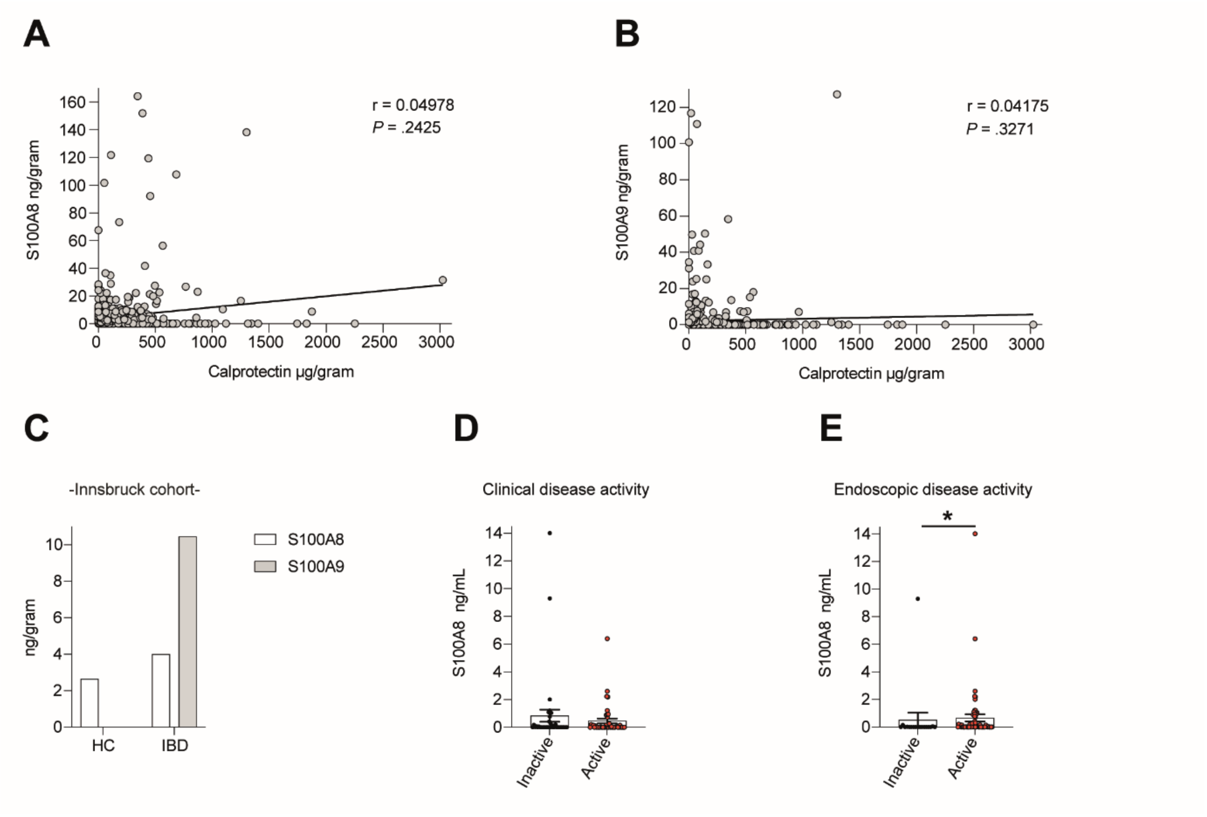

Supplementary Figure 2. Quantification of S100A8 and S100A9 in IBD. A, B. Correlation between fecal S100A8 concentration with fecal calprotectin concentration (**A**) and fecal S100A9 concentration with fecal calprotectin concentration (**B**), as assessed by nonparametric Spearman correlation. Each dot represents one patient from the Innsbruck and 1000IBD Groningen cohort (n=539). **C.** Quantification of fecal S100A8 and S100A9 concentration from HC and IBD patients in Innsbruck with a positive ELISA. **D, E.** Quantification of S100A8 concentration in endoscopic aspirates from the colon of IBD patients with calprotectin concentration ≤150 µg/mL in IBD patients stratified by clinical disease activity (**D**) or endoscopic disease activity (**E**) in a cohort from Kiel, Germany (n=84). *P <.05.

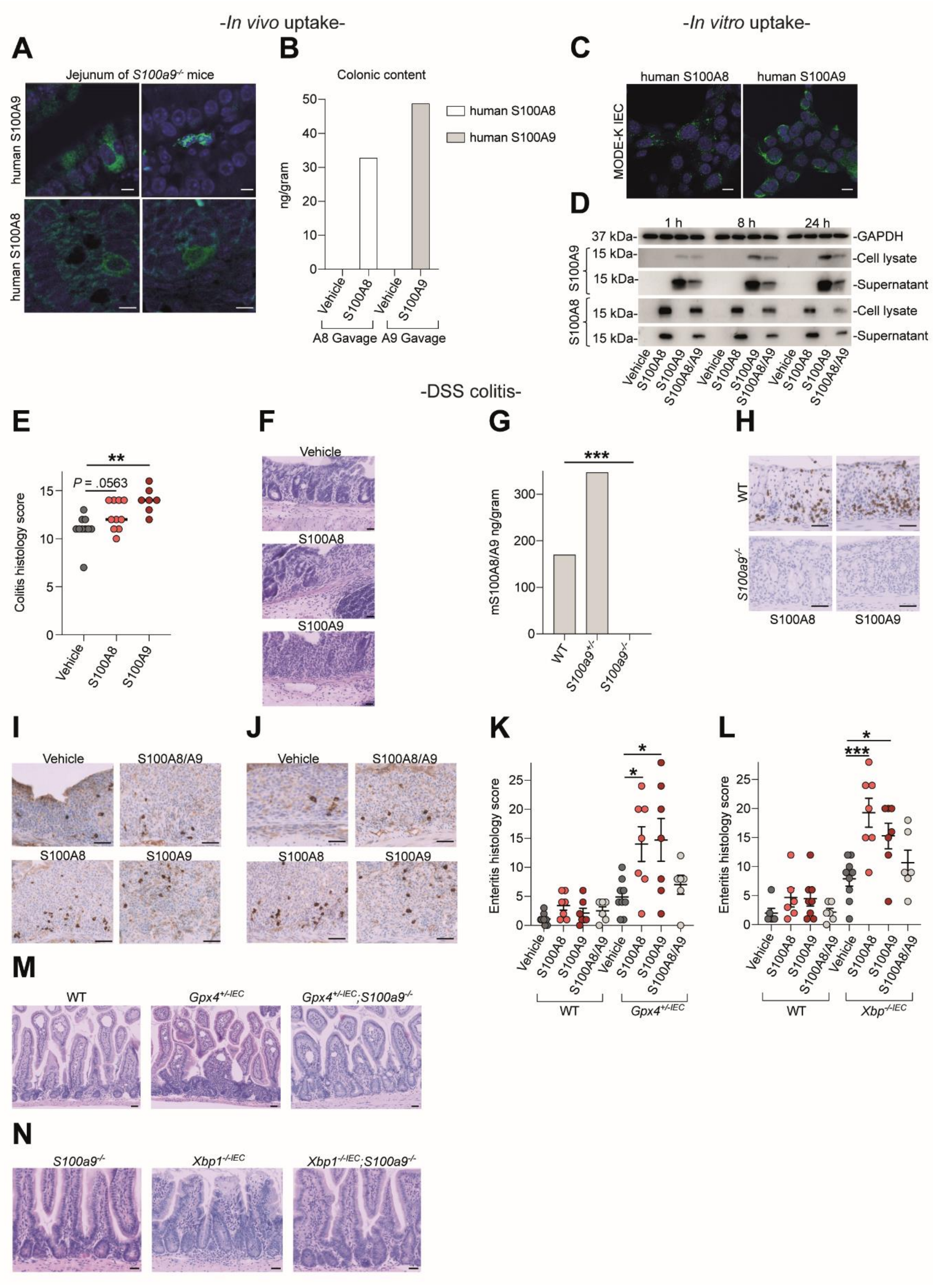

Supplementary Figure 3. Human recombinant S100A8 and S100A9 homodimers promote enteritis and colitis. A. Representative confocal images of the small intestine of *S100a9*$^{-/-}$ mice orally gavaged with human recombinant S100A9 or S100A8 (green) for four days. DAPI (blue) indicates nuclei. Scale bars, 10 µm. **B**. ELISA-based quantification of human S100A8 or human S100A9 in

colonic stool obtained from *S100a9*$^{-/-}$ mice orally gavaged with human recombinant S100A8 or S100A9. **C.** Representative confocal images of MODE-K IECs incubated with human recombinant S100A9 or S100A8 (green) protein. DAPI (blue) indicates nuclei. Scale bars, 5 µm. **D.** Representative immunoblot of human S100A8 and S100A9 from cell lysates and supernatants of MODE-K IECs stimulated with vehicle, S100A8, S100A9 or the 1:1 mix (calprotectin) for indicated time points after rigorous washing. **E, F.** Colitis histology score of DSS (for 5 days) treated *S100a9*$^{-/-}$ mice after vehicle, human S100A8 or S100A9 gavage for 4 consecutive days (**E**), at day 8 (n=7-11; 8-9 weeks; median shown, Kruskal-Wallis-Test with Dunn´s correction) and representative H&E images (**F**). Scale bars, 50 µm. **G, H.** Confirmation of calprotectin deletion in *S100a9*$^{-/-}$ mice by quantification of calprotectin in stool by ELISA (**G**) and by immunohistochemistry of murine S100A8 and S100A9 (**H**) in the colon of mice exposed to DSS. Scale bars, 100 µm. **I, J**. Representative immunohistochemistry images of CD4^{+} T cells (**I**) and CD8^{+} T cells (**J**) in the colon of WT mice exposed to DSS and oral gavage of vehicle or human recombinant S100A8, S100A9 or the 1:1 mix (**as in Figure 3A-F**). Scale bars, 100 µm. **K.** Enteritis histology score of wild-type and *Gpx4*$^{+/-IEC}$ mice fed a PUFA-enriched WD for three months and oral exposure to vehicle or human recombinant S100A8, S100A9 or the 1:1 mix (calprotectin) once daily for the final seven days of the experiment (n=9/7/7/6; 7-8 weeks; mean ± SEM shown; one-way ANOVA with post-hoc Bonferroni). **L.** Enteritis histology score of WT and *Xbp1*$^{-/-IEC}$ mice fed a PUFA-enriched WD for three months and oral exposure to vehicle or human recombinant S100A8, S100A9 or the 1:1 mix (calprotectin) once daily for the final seven days of the experiment (n=9/7/7/6; 7-8 weeks; mean ± SEM shown; one-way ANOVA with post-hoc Bonferroni). **M, N.** Representative H&E images of mice scored in Figure 3L (**M**) and in Figure 3M (**N**). Scale bars, 50 µm. *P <.05, **P <.02, ***P <.001.

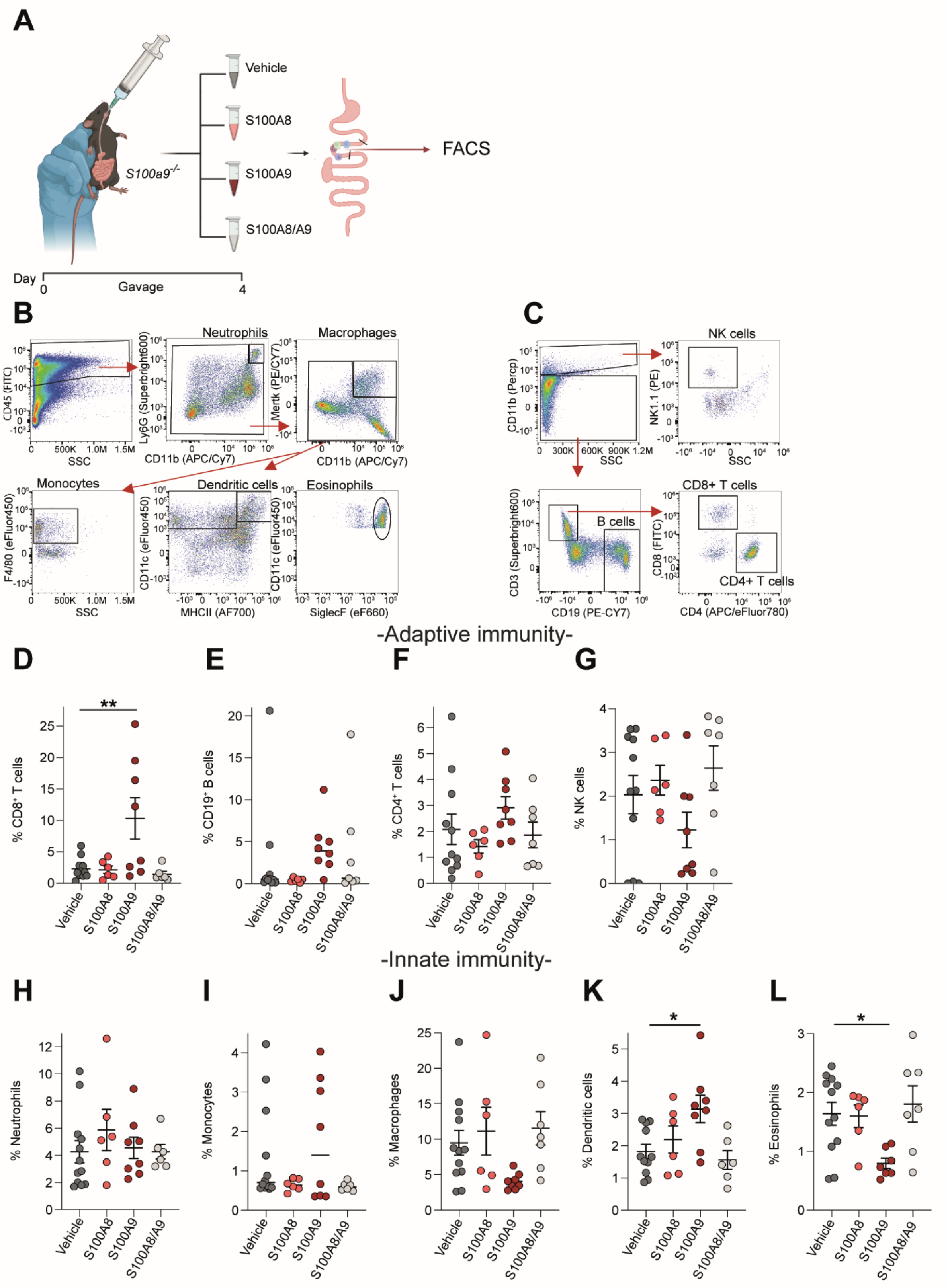

Supplementary Figure 4. Immune phenotyping of mouse gut mucosa after exposure to human S100A8 or S100A9 homodimers. A. Schematic illustration of experimental design: *S100a9*$^{-/-}$ mice were orally gavaged with vehicle or 100 µg S100A8 or S100A9 or the 1:1 mix (calprotectin) once daily for four consecutive days. Tissue samples from small intestinal mucosa (jejunum) were used for flow

cytometry phenotyping. Image was created in https://BioRender.com. **B.** Flow cytometry gating strategy for single $CD45^{+}$, $CD3^{-}$, $CD19^{-}$, $CD49b^{-}$ and $DAPI^{-}$ innate immune cells present in the lamina propria of the small intestine. **C.** Flow cytometry gating strategy for single $CD45^{+}$, $CD11c^{-}$, $F4/80^{-}$, $GR1^{-}$ and $DAPI^{-}$ adaptive immune cells present in the lamina propria of the small intestine. **D-G.** Quantification of $CD8^{+}$ T cells (**D**), $CD19^{+}$ B cells (**E**), $CD4^{+}$ T cells (**F**) and NK-cells (**G**) in *S100a9*$^{-/-}$ mice after oral gavage with vehicle, S100A8, S100A9 or the 1:1 mix (calprotectin) for four days by flow cytometry (n=11/6/8/7; 7-8 weeks; mean ± SEM shown; one-way ANOVA with post-hoc Bonferroni). **H-L.** Quantification of indicated innate immune cells in *S100a9*$^{-/-}$ mice after oral gavage with vehicle, S100A8, S100A9 or the 1:1 mix (calprotectin) for four days (n=12/6/8/7; 7-8 weeks; mean ± SEM shown; one-way ANOVA with post-hoc Bonferroni). Data is depicted in percent of $CD45^{+}$ single cells. *P <.05, **P <.01.

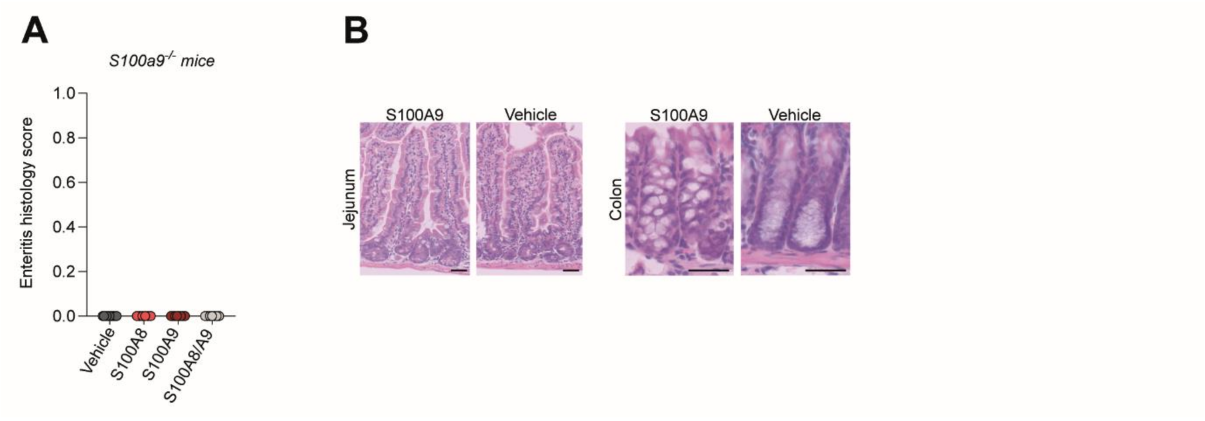

Supplementary Figure 5. S100A8 or S100A9 gavage does not induce gut inflammation. A. Enteritis histology score for *S100a9*$^{-/-}$ mice after oral gavage of vehicle, S100A8, S100A9 or the 1:1 mix (calprotectin) for four days (n=11/6/8/7; 7-8 weeks; median shown). **B.** Representative H&E images of the small intestine and colon of *S100a9*$^{-/-}$ mice after oral gavage with human recombinant S100A9. Scale bars, 50 µm.

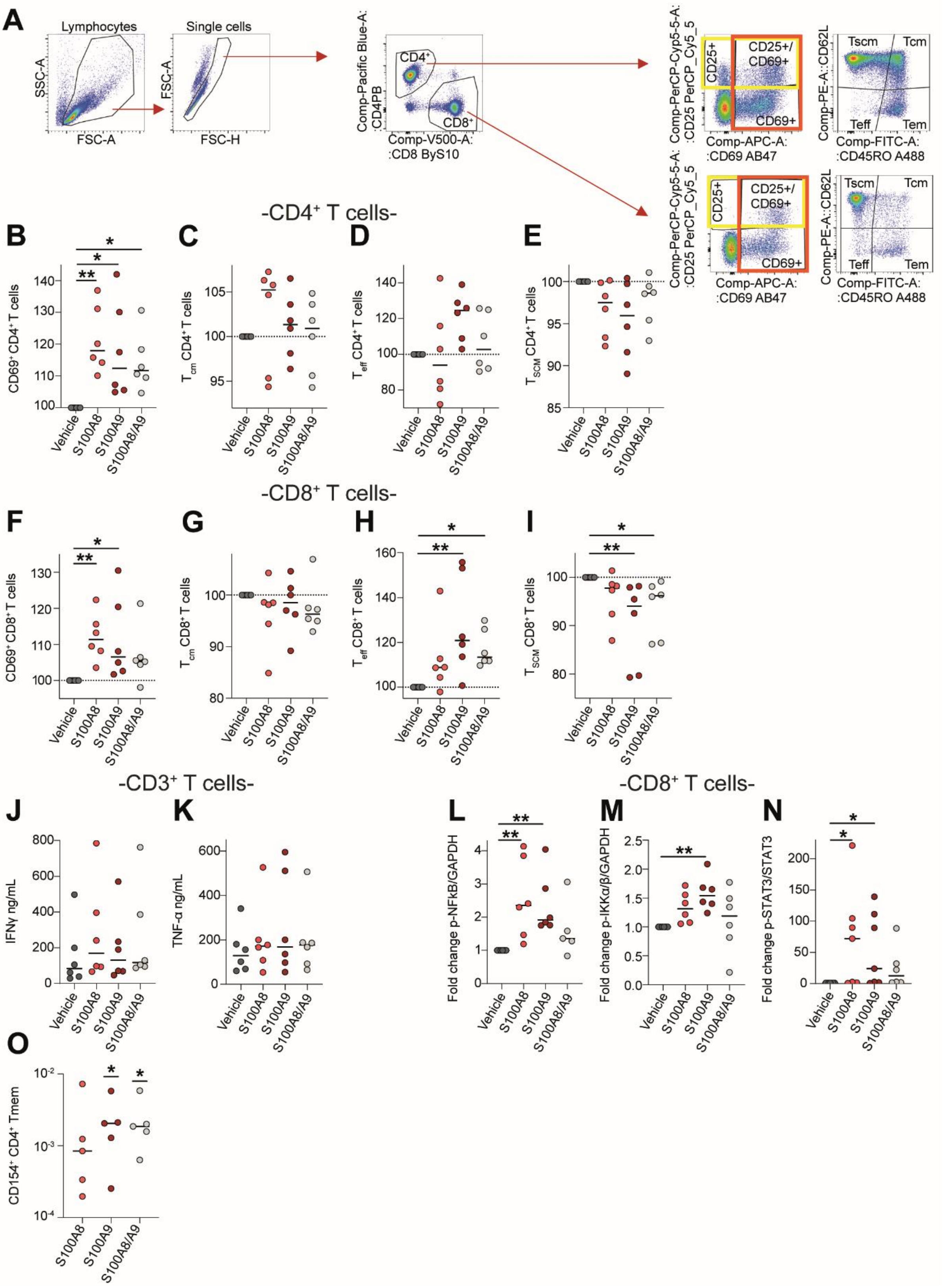

Supplementary Figure 6. Immunophenotyping of human blood-derived CD4$^+$ and CD8$^+$ T cells after stimulation with human recombinant S100A8 and S100A9 homodimers. The experimental approach is summarized in Figure 5A. **A.** Flow cytometry gating strategy for sorted CD3$^+$ T cell that was used to analyze the phenotype of activated CD4$^+$ and CD8$^+$ T cells. Gating was performed for

following surface markers: CD25, CD69, CD62L, CD45RO. Cell populations were defined as follows: T_{scm}: CD62L$^+$/CD45RO$^-$, T_{CM}: CD62L$^+$/CD45RO$^+$, T_{eff}: CD62L$^-$/CD45RO$^-$. **B.** Quantification of activated CD4$^+$ T cells after stimulation with S100A8, S100A9 or the 1:1 mix (calprotectin) for 24 h when compared to vehicle (n=6/6/6/6; Kruskal-Wallis test with Dunn's correction; median shown). **C**. Quantification of central memory CD4$^+$ T cells after stimulation with S100A8, S100A9 or the 1:1 mix (calprotectin) for 24 h when compared to vehicle (n=6/6/6/6; Kruskal-Wallis test with Dunn's correction; median shown). **D**. Quantification of effector CD4$^+$ T cells after stimulation with S100A8, S100A9 or the 1:1 mix (calprotectin) for 24 h when compared to vehicle (n=6/6/6/6; Kruskal-Wallis test with Dunn's correction; median shown). **E**. Quantification of stem cell-like memory CD4$^+$ T cells after stimulation with S100A8, S100A9 or the 1:1 mix (calprotectin) for 24 h when compared to vehicle (n=6/6/6/6; Kruskal-Wallis test with Dunn's correction; median shown). **F.** Quantification of activated CD8$^+$ T cells after stimulation with S100A8, S100A9 or the 1:1 mix (calprotectin) for 24 h when compared to vehicle (n=6/6/6/6; Kruskal-Wallis test with Dunn's correction; median shown). **G**. Quantification of central memory CD8$^+$ T cells after stimulation with S100A8, S100A9 or the 1:1 mix (calprotectin) for 24 h when compared to vehicle (n=6/6/6/6; Kruskal-Wallis test with Dunn's correction; median shown). **H**. Quantification of effector CD8$^+$ T cells after stimulation with S100A8, S100A9 or the 1:1 mix (calprotectin) for 24 h when compared to vehicle (n=6/6/6/6; Kruskal-Wallis test with Dunn's correction; median shown). **I**. Quantification of stem cell-like memory CD8$^+$ T cells after stimulation with S100A8, S100A9 or the 1:1 mix (calprotectin) for 24 h when compared to vehicle (n=6/6/6/6; Kruskal-Wallis test with Dunn's correction; median shown). **J.** Quantification of IFNγ concentration in the supernatant of CD3$^+$ T cells after stimulation with S100A8, S100A9 or the 1:1 mix (calprotectin) for 48 h when compared to vehicle, determined by Bio-Plex (n=6/6/6/6; Kruskal-Wallis test with Dunn's correction; median shown). **K.** Quantification of TNF-α concentration in the supernatant of CD3$^+$ T cells after stimulation with S100A8, S100A9 and S100A8/A9 for 48 h, determined by Bio-Plex (n=6/6/6/6; Kruskal-Wallis test with Dunn's correction; median shown). **L-N.** Quantification of relative phosphorylation of NF-κB (n=6) (**L**), IKKα/β (n=6) (**M**), and STAT3 (n=7) (**N**) by densitometry of immunoblots from human CD8$^+$ T cells stimulated with vehicle, S100A8, S100A9 or the 1:1 mix (calprotectin). **O.** Quantification of antigen-activated CD4$^+$ CD154$^+$ T cells after stimulation with S100A8, S100A9 or the 1:1 mix (calprotectin). Values are shown relative to vehicle stimulation, as is statistical significance (n=5/5/5/5; Kruskal-Wallis test with Dunn's correction; median shown). *P <.05, **P <.01.

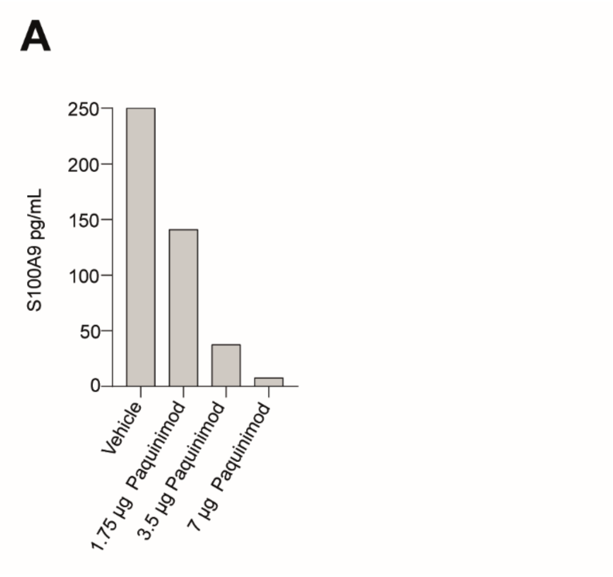

Supplementary Figure 7. Paquinimod inhibits S100A9 suggested by ELISA. A. Quantification of human recombinant S100A9 homodimers in the presence of increasing concentrations of paquinimod, as determined by ELISA.

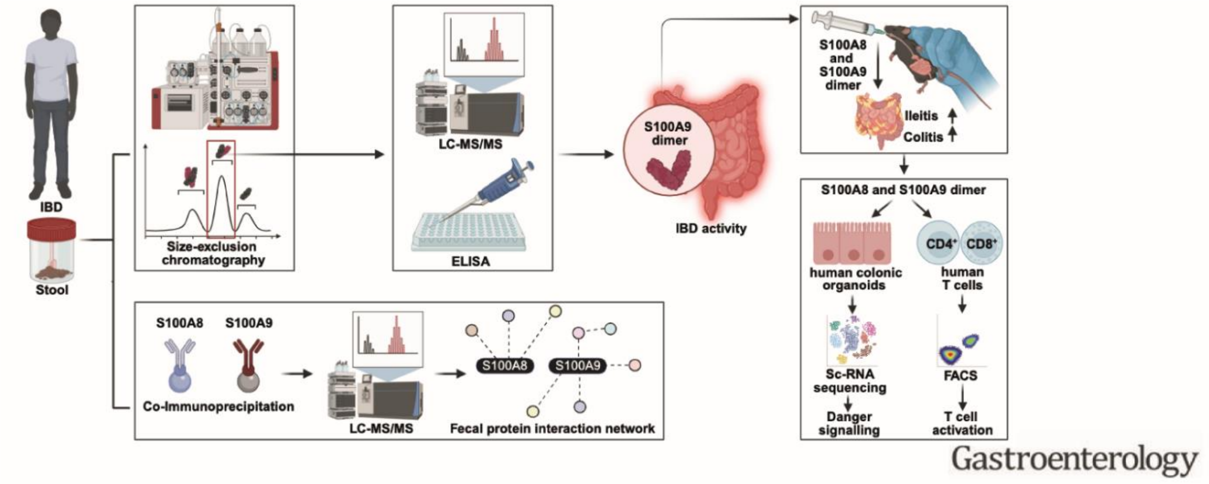

Supplementary Figure 8. Graphical Abstract. Stool and endoscopic aspirates from patients with active IBD commonly contain S100A8 and S100A9 dimers, as validated by size-exclusion chromatography coupled with LC-MS/MS and specific ELISA. Fecal S100A9 detection by ELISA associated with clinical and endoscopic disease activity in IBD patients with low CP concentration (<150µg/g). Human recombinant S100A8 and S100A9 homodimers worsen experimental enteritis and colitis in mice. Human S100A8 and S100A9 homodimers induce epithelial danger signalling in colonic organoids and enhanced activation of $CD4^+$ and $CD8^+$ T cells. In line, adaptive immunity is required for the inflammatory actions of human S100A8 and S100A9 homodimers in the mouse intestine. Moreover, co-immunoprecipitation of S100A8 and S100A9 from stool of patients with IBD coupled with LC-MS/MS identifies the fecal protein interaction network of dimers. Collectively, this study unravels inflammatory actions of S100A8 and S100A9 dimers in IBD which could be used for diagnostic and therapeutic purposes. Image was created in https://BioRender.com.
